# Effects of Home Telemonitoring Interventions on Patients With Chronic Heart Failure: An Overview of Systematic Reviews

**DOI:** 10.2196/jmir.4174

**Published:** 2015-03-12

**Authors:** Spyros Kitsiou, Guy Paré, Mirou Jaana

**Affiliations:** ^1^College of Applied Health SciencesDepartment of Biomedical and Health Information SciencesUniversity of Illinois at ChicagoChicago, ILUnited States; ^2^Research Chair in Information Technology in Health CareDepartment of Information TechnologyHEC MontréalMontréal, QCCanada; ^3^Telfer School of ManagementUniversity of OttawaOttawa, ONCanada

**Keywords:** home telemonitoring, telemedicine, telehealth, remote monitoring, remote consultation, heart failure, chronic diseases, continuity of patient care, physiologic monitoring, ambulatory monitoring, home care services, review, umbrella review, systematic review, meta-analysis

## Abstract

**Background:**

Growing interest on the effects of home telemonitoring on patients with chronic heart failure (HF) has led to a rise in the number of systematic reviews addressing the same or very similar research questions with a concomitant increase in discordant findings. Differences in the scope, methods of analysis, and methodological quality of systematic reviews can cause great confusion and make it difficult for policy makers and clinicians to access and interpret the available evidence and for researchers to know where knowledge gaps in the extant literature exist.

**Objective:**

This overview aims to collect, appraise, and synthesize existing evidence from multiple systematic reviews on the effectiveness of home telemonitoring interventions for patients with chronic heart failure (HF) to inform policy makers, practitioners, and researchers.

**Methods:**

A comprehensive literature search was performed on MEDLINE, EMBASE, CINAHL, and the Cochrane Library to identify all relevant, peer-reviewed systematic reviews published between January 1996 and December 2013. Reviews were searched and screened using explicit keywords and inclusion criteria. Standardized forms were used to extract data and the methodological quality of included reviews was appraised using the AMSTAR (assessing methodological quality of systematic reviews) instrument. Summary of findings tables were constructed for all primary outcomes of interest, and quality of evidence was graded by outcome using the GRADE (Grades of Recommendation, Assessment, Development, and Evaluation) system. Post-hoc analysis and subgroup meta-analyses were conducted to gain further insights into the various types of home telemonitoring technologies included in the systematic reviews and the impact of these technologies on clinical outcomes.

**Results:**

A total of 15 reviews published between 2003 and 2013 were selected for meta-level synthesis. Evidence from high-quality reviews with meta-analysis indicated that taken collectively, home telemonitoring interventions reduce the relative risk of all-cause mortality (0.60 to 0.85) and heart failure-related hospitalizations (0.64 to 0.86) compared with usual care. Absolute risk reductions ranged from 1.4%-6.5% and 3.7%-8.2%, respectively. Improvements in HF-related hospitalizations appeared to be more pronounced in patients with stable HF: hazard ratio (HR) 0.70 (95% credible interval [Crl] 0.34-1.5]). Risk reductions in mortality and all-cause hospitalizations appeared to be greater in patients who had been recently discharged (≤28 days) from an acute care setting after a recent HF exacerbation: HR 0.62 (95% CrI 0.42-0.89) and HR 0.67 (95% CrI 0.42-0.97), respectively. However, quality of evidence for these outcomes ranged from moderate to low suggesting that further research is very likely to have an important impact on our confidence in the observed estimates of effect and may change these estimates. The post-hoc analysis identified five main types of non-invasive telemonitoring technologies included in the systematic reviews: (1) video-consultation, with or without transmission of vital signs, (2) mobile telemonitoring, (3) automated device-based telemonitoring, (4) interactive voice response, and (5) Web-based telemonitoring. Of these, only automated device-based telemonitoring and mobile telemonitoring were effective in reducing the risk of all-cause mortality and HF-related hospitalizations. More research data are required for interactive voice response systems, video-consultation, and Web-based telemonitoring to provide robust conclusions about their effectiveness.

**Conclusions:**

Future research should focus on understanding the process by which home telemonitoring works in terms of improving outcomes, identify optimal strategies and the duration of follow-up for which it confers benefits, and further investigate whether there is differential effectiveness between chronic HF patient groups and types of home telemonitoring technologies.

## Introduction

Heart failure (HF) is a chronic and life-threatening condition that places a substantial burden on health care systems worldwide with high rates of hospitalizations, readmissions, and outpatient visits [1]. Approximately 1-2% of the adult population in the western world has HF, with the prevalence rising to ≥10% among persons 65 years of age or older [2]. Developed countries devote 1%-2% of health care expenditures towards HF, while in the United States alone the estimated direct and indirect annual cost of HF was approximately US $39.2 billion in 2010 [3,4]. Improving the management of the ever-growing population of patients with HF is a high and growing priority for cardiovascular health services.

Home telemonitoring (HT) is a form of non-invasive, remote patient monitoring that has gained attention as a promising strategy to improve the care and management of patients with chronic HF. It can be particularly helpful for those living in remote and rural areas, the elderly and frail who are housebound, as well as those at high risk of deterioration [5]. HT involves the use of electronic devices and telecommunication technologies (eg, monitoring devices, hand-held or wearable technologies, and intelligent sensors) for the digital transmission of physiological and other disease-related data from the patient’s home to a health care center providing care and clinical feedback. By allowing clinical data to be remotely collected on a regular basis, HT can enable early detection of clinical decompensation in patients with HF, allowing for timely intervention to prevent mortality events or further deterioration of the patient’s condition necessitating hospitalization and use of more resources.

HT has attracted a large amount of research over the years both in the form of primary studies and systematic reviews. The extant literature contains published results from numerous trials investigating the clinical, structural, behavioral, or economic effects of HT interventions on patients with chronic diseases [6]. Similarly, the number of published systematic reviews aimed at summarizing the available evidence from primary studies on HT has increased considerably [7-9]. A recent critical review indicated that among all chronic conditions, HF has attracted the highest number of primary studies and systematic reviews [7]. Growing interest on the effects of home telemonitoring on patients with HF has led to a rise in the number of reviews addressing the same or very similar research questions with a concurrent increase in discordant findings in terms of direction and magnitude of HT effects. Differences in scope, methods of analysis, results, and quality of systematic reviews can cause great confusion and make it difficult for policy makers to access the review-level evidence, and for researchers to know where gaps in the evidence exist. Overviews of systematic reviews are an efficient way to gather and summarize in a single source the best available evidence on the effectiveness of interventions. They serve as a useful starting point for decision makers to unpack the evidence towards finding solutions to improve practice and identify areas where new research is needed [10].

This overview aims to collect, appraise, and summarize evidence from multiple systematic reviews examining the effects of HT interventions on patients with HF with a view to providing policy makers and practitioners with the evidence they need to make informed decisions related to the telemonitoring of HF patients. It also aims to identify research gaps in this area and suggest avenues for future research.

## Methods

### Overview

As shown below, the Cochrane Collaboration methodology [11] and available methodological guidelines for overviews of reviews [12,13] were rigorously applied throughout this study.

### Search Methods

A systematic search of MEDLINE, EMBASE, CINAHL, and the Cochrane Library (Cochrane Database of Systematic Reviews, DARE, and the Health Technology Assessment Database) was conducted on December 13, 2013, and updated on December 3, 2014, using key terms and clinical query filters [14,15] to identify all relevant, peer-reviewed systematic reviews of HT interventional studies for patients with chronic HF, published since January 1, 1996. No language restrictions were applied. Details of the full search strategy are presented in Multimedia Appendix 1. Searches were supplemented by manual searching of two relevant scientific journals (ie, *Journal of Telemedicine and Telecare* and *Telemedicine and e-Health*) and screening of relevant reviews’ bibliographies.

### Selection of Systematic Reviews

In the preliminary stage, all titles and abstracts were screened by the first author according to the pre-specified inclusion criteria described in Table 1. References that clearly did not meet all of the criteria were excluded. Full-text articles were then retrieved and independently assessed for inclusion by the first 2 authors. Any disagreements were resolved through discussion. The third author was available for arbitration in case of persistent disagreements. Reviews were excluded if they studied the effects of HT on patients with other chronic or long-term conditions but did not report findings for HF separately.

**Table 1:** Inclusion criteria for the selection of relevant systematic reviews.

Criteria categories	Description of inclusion criteria
Study type	Systematic reviews (with or without meta-analysis) of original, interventional studies. Following the definitions used by the Cochrane Collaboration and the Preferred Reporting Items for Systematic Reviews and Meta-Analyses (PRISMA) statement, a systematic review was defined as a review that attempts to search, identify, appraise, and collate all empirical evidence that fits pre-specified eligibility criteria to answer a clearly stated set of objectives or specific research question(s), using explicit and systematic methods with a view to minimizing risk of bias.
Publication type	Full, peer-reviewed articles published in English.
Population	Patients with definitive diagnosis of HF.
Intervention	HT defined as the use of non-invasive devices in conjunction with information and communication technologies to monitor and electronically transmit physiological, biometric, and/or disease-related data (eg, arterial blood pressure, weight, cardiac rate, medications, symptoms) from the patient at home to the health care provider responsible for monitoring remotely the patient’s health status.
Comparisons	Standard (usual) care or other non–home telemonitoring approaches.
Outcomes	Primary or secondary outcomes pertaining to the clinical, structural, behavioral, or economic effects of HT. More specifically, systematic reviews reporting at least one of the following outcomes and having met the abovementioned criteria were eligible for inclusion: mortality, all-cause hospitalizations, HF-related hospitalizations, emergency department visits, clinic/outpatient visits, quality of life, cost-effectiveness, patient satisfaction, acceptability, and compliance/adherence.

### Data Extraction

Data relating to key characteristics of the reviews, including information about the objectives, participants, intervention features, outcomes assessed, and comparisons performed; as well as the quality of included studies, quality of the reviews, pooled effect sizes for outcomes meta-analyzed, and main conclusions were extracted using an electronic form that was developed for the purposes of this review. Data were extracted by the first author and were verified for accuracy by the second. Differences were resolved in group meetings.

### Quality Assessment of Included Reviews

The methodological quality of the included systematic reviews was independently assessed by the first two authors using the Assessment of Multiple Systematic Reviews (AMSTAR) tool [16]. AMSTAR is a validated instrument that uses 11 items to assess the degree to which review methods are unbiased. To ensure consistency of assessment between the 2 assessors, we developed decision support rules for scoring each criterion (Multimedia Appendix 2). Based on the results of the critical appraisal, reviews were categorized into three categories: “low” (score 0 to 3); “middle” (score 4 to 7); and “upper” (score 8 to 11). These groups reflect the existence of “major”, “moderate”, and “minor or no methodological limitations” in the included reviews, respectively.

### Quality of Evidence in Included Systematic Reviews

Quality of evidence in included systematic reviews was assessed by outcome using the Grades of Recommendation, Assessment, Development, and Evaluation (GRADE) system criteria [17-27]. GRADE identifies five key elements that influence quality of evidence and can be used for rating down one’s confidence in the estimates of intervention effects. These are (1) risk of bias (limitations in the design and execution of primary studies included in the reviews) [20], (2) inconsistency (statistical heterogeneity between estimates of effect across studies) [23], (3) indirectness (applicability of participants, interventions, and outcomes to the clinical question under consideration) [24], (4) imprecision (impact of random error as reflected by the relative confidence interval of the summary effect estimate) [22], and (5) publication bias (publication or non-publication of research findings depending on the direction and statistical significance of the results of the primary studies) [21]. Assessing and combining these components determine the quality of evidence for each outcome of interest as “high” (ie, further research is very unlikely to change our confidence in the estimate of effect), “moderate” (ie, further research is likely to have an important impact on our confidence in the estimate of effect and may change the estimate), “low” (ie, further research is very likely to have an important impact on our confidence in the estimate of effect and is likely to change the estimate), or “very low” (ie, any estimate of effect is very uncertain). In cases where reviews had not performed a risk of bias assessment, we performed this task independently using the six criteria of the Cochrane Collaboration tool [28]. This facilitated application of the GRADE system and development of standardized summary of findings tables by outcome [11,29], as explained below, using the GRADEpro software (version 3.6). When a review had performed risk of bias and quality of evidence assessments, we collected this information during the data extraction process.

### Analysis and Synthesis

To summarize the evidence on the effectiveness of HT interventions, we designed a 3-step process [30,31]. First, as a means of evaluating the comparability of the included systematic reviews and the extent to which reviews overlapped in terms of included studies, we carried out a bibliographic analysis that cross-linked individual systematic reviews with cited HT studies (Multimedia Appendix 3). Following the methodology used in Martel et al [32], we then calculated the ratio of cited to total pre-existing HT studies for each systematic review, taking into account the lag time between the reported end-date of search for identification of interventional studies and the actual publication date of the review. The mean ratio and 95% confidence intervals (CI) were calculated for both randomized controlled trials (RCTs) and observational studies to reflect the overall degree of overlap between the reviews. Second, we closely examined the objectives, participants, interventions, comparisons, and outcomes (PICO) characteristics of each review and subsequently categorized the reviews into homogeneous groups according to their common elements and relevant comparisons. We then recalculated the ratio of cited to total HT primary studies in order to evaluate the degree of overlap between reviews in the same group. These two steps were designed with a view to identify differences across reviews in terms of scope, range of included studies, and ways that HT interventions were split or lumped together, and to allow us formulate meaningful interpretation of the extracted data and results by disaggregating the evidence as appropriate. Third, within each group of the developed classification scheme, we ranked the reviews according to their methodological quality (AMSTAR score), documented the consistency of findings and conclusions across all reviews, and constructed standardized summary of findings tables following the Cochrane Collaboration guidelines [11,29] to summarize the effects of HT interventions from the most direct evidence (ie, from reviews that achieved the highest methodological quality score in each group of the taxonomy). In presenting the effects of HT, we focused on outcomes that were reported in more than 50% of the systematic reviews.

## Results

### Description of the Included Systematic Reviews

**Figure 1:**
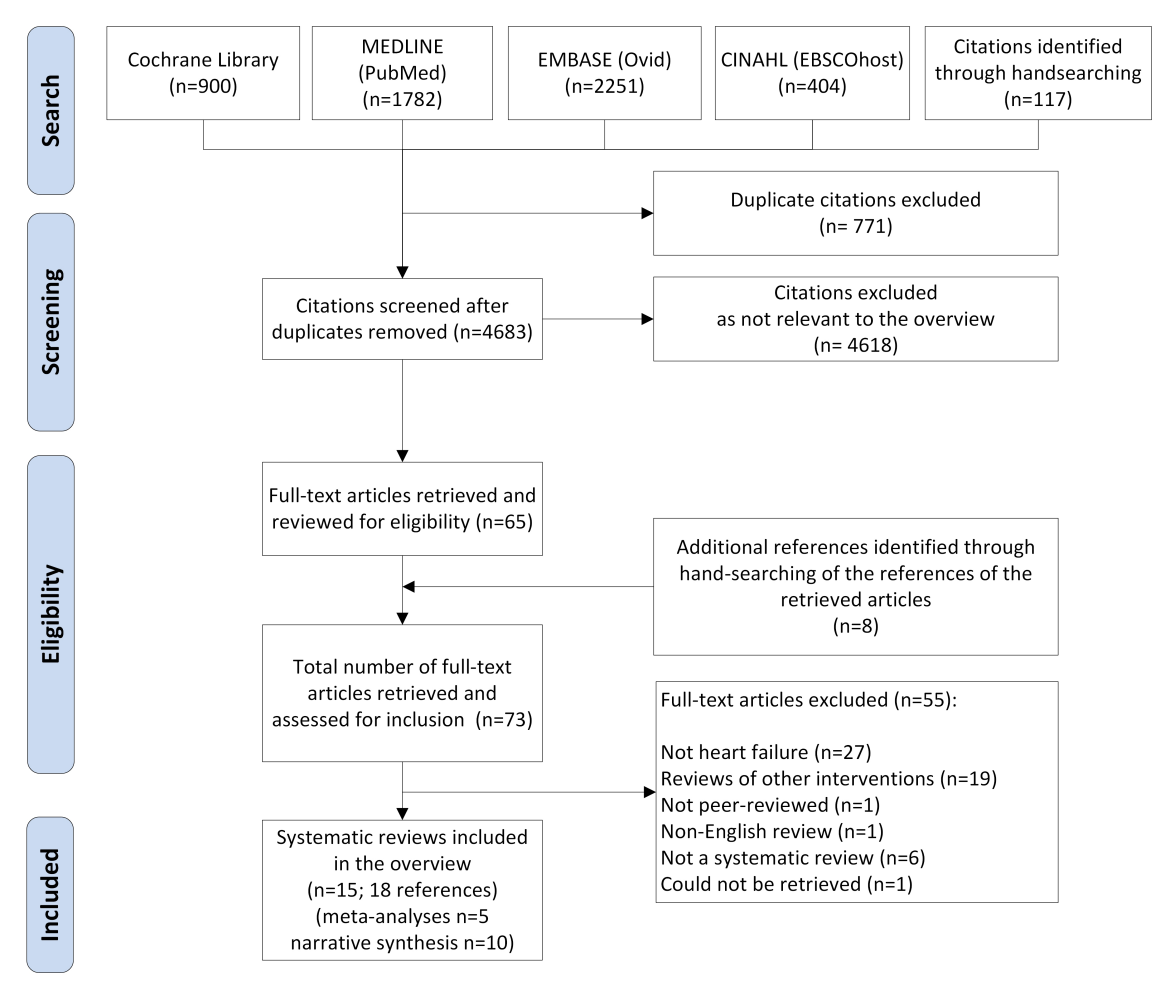
Selection process of the systematic reviews.

As shown in Figure 1, our search (up to December 13, 2013) yielded 4683 citations after removal of 771 duplicate references. After screening titles and abstracts, we retrieved 65 reviews in full text for further assessment. The references of these articles were manually screened to identify any relevant reviews that were not originally captured by our search strategy. This process yielded 8 more reviews. After further assessment, we excluded 55 articles that did not meet our eligibility criteria. Details about the primary reasons for exclusion and the full references of the excluded articles are provided in Multimedia Appendix 4. After completion of the screening process, we identified 15 systematic reviews for inclusion (18 references; 3 systematic reviews had duplicate publications) [33-50].

General characteristics about the population, interventions, and comparison groups included in the 15 systematic reviews along with the main conclusions of each review are summarized in Table 2. Reviews were published between 2003 and 2013, with more than half (n=9) published in 2009 or later. All reviews were published in peer-reviewed journals. However, two systematic reviews were initially published as Health Technology Assessment reports and one as a Cochrane review. Five reviews (33%) contained meta-analysis for at least one primary outcome of interest, while the remaining 10 used narrative synthesis. Seven reviews (based on the corresponding author) originated in North America (Canada=5 and United States=2), 6 in Europe (United Kingdom=3, Greece=1, Netherlands=1, Spain=1), and 2 in Australia. Four systematic reviews (27%), in addition to HT investigated the effects of structured telephone support (STS) on patients with HF [33,34,37,41]. However, findings from these distinctively different interventions were analyzed and reported separately. Therefore, data extracted and presented in Table 2 from these reviews pertain only to the HT studies and not the STS ones.

The number of HT studies identified and included in the reviews ranged from 4 to 42: mean 17.53 (SD 11.4). The total number of identified studies was 105; 38 (36%) referring to RCTs and 67 (64%) to observational studies. The citation patterns of these publications are presented in Multimedia Appendix 3. Seven reviews (47%) included only RCTs [33,34,36,37,41,43,47]. The remaining reviews included studies with quasi-experimental and cohort designs. The overall degree of overlap between the included reviews expressed as the mean ratio of cited to total pre-existing HT studies was 0.40 (95% CI 0.29-0.52) for RCTs, ranging from 0.11-1.00, and 0.25 (95% CI 0.1-0.42) for observational studies, ranging from 0.08-0.50.

**Table 2:** Characteristics of included systematic reviews.

Authors (year)	Years searched	Number and design of HT studies	Population (mean age; disease severity)	Intervention (length of follow-up)	Control group	Main conclusions
Clark et al (2007) [33]	2002 to May 2006	5 RCTs	807 patients (mean age range 57-75; NYHA class I-IV)	HT without home visits (Follow-up: 3-16 months)	Usual care	HT reduced all-cause mortality and HF-related hospitalizations
						Results were mixed for quality of life and costs
Inglis et al, 2010 [34,48]	2002 to Nov. 2008	14 RCTs	2710 patients (mean age range 57-78 years; NYHA class I-IV; most II-IV)	HT without home visits (Follow-up: 3-15 months)	Usual care	HT reduced the risk of all-cause mortality and HF-related hospitalizations
						HT improved quality of life and reduced costs
						No consistent impact on length of stay
Polisena et al, 2010 [35,49]	1998-2008	21 studies (11 RCTs, 10 observational)	3082 patients (mean age range 52-79; NYHA class I-IV; most III-IV)	HT with or without home visits (Follow-up: 1-12 months)	Usual care	HT reduced mortality and hospitalizations
						Patient quality of life with HT was similar or better than with usual care
Clarke et al, 2011 [36]	1969 to Oct. 2009	13 RCTs	3480 patients (mean age range 55-85 years; NYHA class I-IV)	HT interventions with or without home visits (Follow-up: 3-15 months)	Usual care	HT reduced all-cause mortality and HF hospitalizations
						HT in conjunction with nurse home visiting and specialist unit support can be effective in the clinical management of patients with HF and help improve their quality of life
Pandor et al, 2013 [37,50]	2002 to Jan. 2012	20 RCTs [10 RCTs of recently discharged patients (≤28 days) + 10 RCTs of patients with stable HF]	6561 patients [1918 recently discharged patients (mean age range 57-78 years; NYHA class: I-IV; most II-IV); 4643 patients with stable HF (mean age not summarized; NYHA class: I-IV)]	HT without home visits using patient-initiated external electronic devices with transfer of physiological data from the patient to the health care provider by landline or mobile phone, cable network or broadband technology (Follow-up: 3-12 months, recently discharged patients; 6-22 months, patients with stable HF)	Usual care	HT with medical support provided during office hours showed beneficial trends in reducing all-cause mortality for recently discharged patients with HF. However, these effects were statistically inconclusive
						Where usual care is below average or suboptimal, the impact of remote monitoring is likely to be greater
Louis et al, 2003 [38]	1966-2002	24 studies (6 RCTs, 12 observational)	2629 patients (mean age range 53-82 years; NYHA class: I-IV; most II-IV)	HT of patients using special telecare devices in conjunction with a telecommunication system (Follow-up: 2-18 months)	Usual care, home visits, and/or nurse telephone support	HT improved mortality, yet adequately powered multicenter RCTs are required to further evaluate the potential benefits and cost-effectiveness of this intervention
Martínez et al, 2006 [39]	1966 to April 2004	42 studies (13 reports of 10 RCTs, 29 observational)	2303 patients (5 studies did not specify number of participants) (mean age range 48-83; NYHA class I-IV; most II-IV)	HT using peripheral devices for measuring and automatically transmitting physiological data (Follow-up: 1-24 months)	Usual care, home nurse visits, pre/post HT	Reduces hospital readmissions, length of stay, mortality, emergency visits, and costs
						It is viable, easy to use, and is widely accepted by patients and health professionals
Paré et al, 2007 [40]	1990-2006	16 studies (7 reports of 5 RCTs, 9 observational)	Not summarized	HT as an automated process for the transmission of patient health status data (Follow-up: 1 to 36 months)	Usual care, home visits, pre/post HT	Promising patient management
						Future studies need to build evidence related to its clinical effects, cost effectiveness, impacts on services utilization, and acceptance by health care providers
Chaudhry et al, 2007 [41]	1966 to Aug. 2006	4 RCTs	774 patients with HF (mean age range 59-70 years; NYHA class I-IV)	HT with or without home visits (Follow-up: not summarized)	Usual care, home visits	HT may be an effective strategy for disease management in high-risk heart failure patients , but the evidence base is currently quite limited
Seto 2008 [42]	up to April 2007	10 studies (5 RCTs, 4 observational, 1 survey)	1394 patients with HF (mean age range 58-74 years; NYHA not summarized)	HT with a component of home physiological measurement (Follow-up: 2-36 months)	Usual care, home visits, pre/post HT	All studies found cost reductions (range: 1.6% to 68.3%) mostly related to reduced hospitalization expenditures
Dang et al, 2009 [43]	1966 to Apr. 2009	9 RCTs	2020 adult patients with HF (mean age range 53-79 years; NYHA class II-IV)	Home telehealth remote monitoring (ie automated or physiologic monitoring of signs and symptoms; two-way video monitoring with or without physiologic monitoring; Internet, Internet Protocol, or Web-based technologies or image capture and transfer) (Follow-up: 3-12 months)	Usual care, home visits	Telemonitoring is a promising strategy.
						More research required to determine the ideal patient population, technology, and parameters, frequency and duration of telemonitoring, and the exact combination of case management and close monitoring that would assure consistent and improved outcomes with cost reductions in HF
Maric et al, 2009 [44]	Up to Aug. 2007	42 studies; 52 references (12 RCTs, 30 observational)	4290 patients (9 studies did not specify number of participants) (mean age and NYHA class not summarized)	HT using modalities that transmit data to health care professionals to assist in self-monitoring (eg, telephone-based touch pad, website based modalities, video consultations, and other technology-assisted devices) (Follow-up: 1-18 months)	Usual care, home visits, nurse telephone support, pre/post HT	Most studies demonstrated improvements in outcome measures, including improved QoL and decreased hospitalizations. However, not all studies reported the same improvements and in several cases the sample sizes were relatively small
Paré et al, 2010 [45]	1966-2008	17 studies (13 reports of 10 RCTs, 4 observational)	Not summarized	HT interventions in which physiological and biological data are transferred from the patients’ home to the telemonitoring center to monitor patients, interpret the data, and make clinical decisions (Follow-up: not summarized)	Usual care, home visits, pre/post HT	Many studies failed to show a reduction in either mortality or hospitalization rates, although a few trials have reported a trend towards shorter lengths of stay in hospital.
						Due to the equivocal nature of current findings of HT involving patients with HF, larger trials are still needed to confirm the clinical effects of this technology for these patients.
Kraai et al, 2011 [46]	Up to November 2010	14 studies (4 RCTs, 10 observational)	2005 patients (mean age range 50-78; NYHA not summarized)	Noninvasive remote monitoring with external equipment to measure physiologic data such as weight and blood pressure (Follow-up: not summarized)	Usual care, home visits, nurse telephone support, pre/post HT	In general, patients seemed to be satisfied or very satisfied with HT
Giamouzis et al, 2012 [47]	2001 to Nov. 2011	12 RCTs	3877 patients (mean age range 57-78; NYHA class I-IV; most II-IV)	HT with at least one device that measured physiological data provided by the researchers for home use (Length of follow-up: 6 to 26 months)	Usual care	Currently available trial results tend to be in favor of HT
						HT was highly acceptable by HF patients

### Population

As shown in Table 2, the majority of systematic reviews reported the mean age (12/15, 80%) and New York Heart Association (NYHA) class of the participants (10/15, 67%). The highest reported mean age in a study was 85 years [36] and the lowest was 48 years [39]. The NYHA class of participants in all but one review ranged from I to IV. Six reviews reported that most participants in the included studies were NYHA class II-IV [34-35,37-39,47]. One review [37] focused primarily on HF patients who had been recently discharged (within 28 days) from an acute care setting after a recent HF exacerbation and conducted additional analyses to assess whether or not the results from these studies differed markedly from the results of studies that included patients with stable HF. The remaining reviews did not make this distinction and combined in their analysis studies with both recently discharged and stable HF patients.

### Intervention

Reviews consisted of a family of complex HT interventions, rather than a single type, involving various telehealth devices (eg, videoconferencing equipment, automated telemonitoring stations, mobile phones, and interactive voice or symptom response systems), technological approaches for data collection and transmission (eg, modem, broadband, or mobile phone transmission; Web-based or telephone touch-pad data entry), as well as other chronic disease management strategies (eg, education and home visits). However, reporting of the active ingredients of these interventions was often poor in the included reviews and not consistently performed for all primary studies (eg, [36,38-40,46,47]). While the majority of systematic reviews (10/15, 67%) summarized and reported relevant information about the type of physiological measures monitored in the included trials [33,34,37,38,41-44,47,49], and in fewer cases the frequency with which data transmission or communication between the patients and clinicians occurred [33,34,37,41,43,49], most reviews (8/15, 53%) did not provide sufficient details about the technology used in each of the included primary studies [33,34,36-40,45-47] (Table 3). Furthermore, virtually all reviews treated HT as a “black box” making no attempts to investigate whether technological differences between HT interventions are associated with different effects. In five reviews [37,38,45-47], the description of the intervention extracted from the trials was reduced into just a few words presented in a summary table or within the text, while three reviews did not provide any information at all about the included interventions [36,39-40]. Also, out of the 15 systematic reviews, only two classified and analyzed HT interventions according to the technologies used [41,44]. Lack of sufficient details about the included interventions coupled with the way that systematic reviews have analyzed the effects of HT up to now, make it difficult to determine what types of HT technologies have been used in the primary studies included in the systematic reviews, what proportion of studies included in the systematic reviews have used each type of HT technology, and more importantly, which HT technologies are more effective. We think this issue deserves more attention. This is why we have decided to extract additional data and conduct post-hoc analyses. Results are presented and discussed at the end of this section.

**Table 3:** Characterization of HT technologies in systematic reviews.

Author (year)	Does the review present information from all studies about the types of HT technologies used in the intervention group?	Does the review present information from all studies about the types of physiological parameters monitored in the intervention group?	Does the review classify and analyze studies according to the different types of HT technologies?
Inglis et al (2010) [34]	Yes	Yes	No
Clark et al (2007) [33]	Yes	Yes	No
Giamouzis et al (2012) [47]	No	Yes	No
Pandor et al (2013) [37]	No	Yes	No
Polisena et al (2010) [35]	Yes	Yes	No
Clarke et al (2011) [36]	No	No	No
Chaudhry et al (2007) [41]	Yes	Yes	Yes
Dang et al (2009) [43]	Yes	Yes	No
Louis et al (2003) [38]	No	Yes	No
Martinez et al (2006) [39]	No	No	No
Paré et al (2010) [45]	No	No	No
Kraai et al (2011) [46]	No	No	No
Maric et al (2009) [44]	Yes	Yes	Yes
Seto (2008) [42]	Yes	Yes	No
Paré et al (2007) [40]	No	No	No

### Comparison

Six systematic reviews included RCTs in which the control group received usual (routine) care. This involved provision of post-discharge multidisciplinary care without intensified follow-up or home visits. Usual care varied across studies from in-person follow-up visits to a general practitioner or primary care provider to attendance at an outpatient clinic-based disease management program [33,34,36,37,47,49]. The remaining reviews, in addition to usual care, included RCTs that compared HT with other relevant comparators (eg, home visits or telephone support).

### Outcomes

Despite differences in the scope and range of included studies, most reviews reported on a number of similar outcomes (Table 4). Most frequently reported outcomes included all-cause mortality (11/15, 73%), hospital re-admissions (13/15, 87%), costs (12/15, 80%), and length of stay (9/15, 60%). Six reviews made a distinction between all-cause and HF-related hospitalizations, while the remaining reviews did not. This methodological deficiency gave rise to unit-of-analysis errors, as authors often mixed study results from these two different outcomes. Other commonly reported outcomes comprised the impact of HT interventions on health care costs, quality of life, and length of stay in hospital. Compliance, acceptability, emergency room visits, and patient satisfaction were reported in less than half of the reviews and thus were excluded from our analysis.

**Table 4:** Outcomes reported by the systematic reviews^a^.

Author (year)	ACM	ACH	HFH	HOSP	Costs	QoL	LoS	CA	ACC	ER	PS	OV
Inglis et al (2010) [34]	X	X	X		X	X	X	X	X			
Clark et al (2007) [33]	X	X	X		X	X		X	X			
Giamouzis et al (2012) [47]	X			X	X							
Pandor et al (2013) [37]	X	X	X			X	X	X	X		X	
Polisena et al (2010) [35]	X	X	X			X	X			X	X	X
Clarke et al (2011) [36]	X	X	X		X	X	X	X	X	X		
Chaudhry et al (2007) [41]	X	X	X		X							
Dang et al (2009) [43]	X	X	X		X		X			X		X
Louis et al (2003) [38]	X			X	X			X	X			
Martinez et al (2006) [39]	X			X	X	X	X		X			
Paré et al (2010) [45]	X			X			X					
Kraai et al (2011) [46]											X	
Maric et al (2009) [44]				X	X	X				X		
Seto (2008) [42]					X							
Paré et al (2007) [40]				X	X	X	X	X		X		

^a^ACM: all-cause mortality; ACH: all-cause hospitalizations; HFH: HF-related hospitalizations; HOSP: Hospitalizations (indicates reviews that did not make a distinction between all-cause and HF-related hospitalizations); Costs: Cost Savings; QoL: Quality of life; LoS: Length of stay; CA: Compliance; ACC: Acceptability; ER: Emergency room visits; PS: Patient satisfaction; OV: Outpatient visits.

### Methodological Quality of Included Systematic Reviews

The methodological quality of the reviews varied considerably, and many of them had important limitations (Table 5). For example, only 5 reviews assessed risk of bias in the primary studies (Q7), while quality of evidence (Q8) was used in the interpretation of results in just four reviews. Only three reviews were found to be of high quality, achieving a score of 8 or more on the 11-point AMSTAR scale (3/15, 20%). These reviews indicated minimal bias in their design and execution. Four reviews were of moderate quality, scoring between 4 and 7 points, and eight reviews were of low quality scoring less than 4 points. Of the moderate quality reviews, most received a rating of 5 or less, which suggests that these reviews along with the ones of low quality may be at risk of important bias.

**Table 5:** Methodological quality of systematic reviews based on AMSTAR criteria and scores.^a,b^

Author (year)	Q1	Q2	Q3	Q4	Q5	Q6	Q7	Q8	Q9	Q10	Q11	Total
Inglis et al (2010) [34]	Y	Y	Y	Y	Y	Y	Y	Y	N	Y	Y	10
Pandor et al (2013) [37]	Y	N	Y	Y	Y	Y	Y	Y	Y	N	Y	9
Polisena et al (2010) [35]	Y	Y	Y	N	N	Y	Y	Y	Y	N	Y	8
Clark et al (2007) [33]	N	Y	Y	Y	N	Y	Y	N	Y	Y	N	7
Chaudhry et al (2007) [41]	N	CA	Y	CA	N	Y	Y	Y	Y	N/A	N	5
Dang et al (2009) [43]	N	CA	Y	N	Y	Y	N	N	Y	N/A	N	4
Paré et al (2010) [45]	Y	Y	N	N	Y	N	N	N	Y	N/A	N	4
Louis et al (2003) [38]	N	CA	Y	Y	N	Y	N	N	N	N/A	N	3
Martinez et al (2006) [39]	N	CA	Y	Y	N	Y	N	N	N	N/A	N	3
Giamouzis et al (2012) [47]	N	CA	Y	N	N	Y	N	N	N	N/A	N	2
Clarke et al (2011) [36]	N	CA	Y	N	CA	N	N	N	N	Y	N	2
Kraai et al (2011) [46]	N	CA	N	N	N	Y	N	N	Y	N/A	N	2
Maric et al (2009) [44]	N	N	N	N	N	Y	N	N	Y	N/A	N	2
Seto (2008) [42]	N	CA	N	N	N	Y	N	N	Y	N/A	N	2
Paré et al (2007) [40]	N	CA	Y	N	N	N	N	N	N	N/A	N	1

^a^Q1: A priori design; Q2: Duplicate study selection and data extraction; Q3: Search comprehensiveness; Q4: Inclusion of grey literature; Q5: Included and excluded studies provided; Q6: Characteristics of the included studies provided; Q7: Scientific quality of the primary studies assessed and documented; Q8: Scientific quality of included studies used appropriately in formulating conclusions; Q9: Appropriateness of methods used to combine studies’ findings; Q10: Likelihood of publication bias was assessed; Q11: Conflict of interest – potential sources of support were clearly acknowledged in both the systematic review and the included studies.

^b^“Y” (Yes): Criterion met; “N” (No): Criterion not met; CA: Cannot answer; N/A: Not applicable. We awarded one point to each item that scored “yes” and summed these to calculate a total score for each review.

### Classification of Reviews

Although ostensibly the 15 systematic reviews may appear to be similar, a closer examination of their PICO characteristics and criteria used for the selection of HT studies revealed several differences. For example, some reviews had a focused scope of inquiry and used narrow inclusion criteria to examine the effects of HT interventions without home visits for clinical assessment or educational purposes versus usual care. Other reviews had a broader scope of inquiry and examined the effects of HT interventions (with or without home visits) versus a variety of comparison interventions, including usual care, nursing telephone support, and/or home care. To overcome such heterogeneity, we carefully examined the population, intervention(s), and comparison group(s) that each review addressed, and subsequently classified reviews into homogeneous groups. Table 6 presents the taxonomy that was developed and used to facilitate the synthesis process.

One of the reviews [37] incorporated a network meta-analysis to determine the effectiveness of different remote monitoring strategies versus usual care on adult HF patients who had been recently discharged home (≤28 days) from an acute care setting and also (in additional analyses) on patients with stable HF. Remote monitoring strategies included STS, HT with clinical support during offices hours, and HT with clinical support provided 24/7. This review provided evidence for multiple comparison pairs and thus, findings from each comparison that was relevant to our overview were extracted and examined separately. For Comparison 2 (Table 6), it should be noted that we extracted the results of the sensitivity analysis that Pandor et al [37] performed, based on which a study that appeared to be an outlier (because patients in the control group received better-than-usual support and optimal medical treatment) was excluded.

Given the above classification of the reviews (Table 6), the ratio of cited to total pre-existing HT studies was recalculated to evaluate the degree of overlap between reviews in the same comparison group. Comparison groups 2, 3, and 4 were excluded from this analysis because they each comprised only one systematic review. Overall, the analysis yielded a mean ratio of 0.87 (95% CI 0.29-1.44) for RCTs in Comparison group 1, a mean ratio of 0.66 (95% CI 0.43-0.90) for RCTs in Comparison group 5, and a mean ratio of 0.48 (95% CI 0.28-0.67) for RCTs in Comparison group 6. The ratio of cited to total published observational studies remained the same, as virtually all reviews that included studies with observational designs were classified in the same group (Comparison 6). The observed increase in the mean ratio of cited to total pre-existing RCTs in each group compared to the overall mean ratio presented earlier, further supports the theoretically appealing classification of systematic reviews into groups and validates the methodological approach of summarizing the effects of HT interventions from the most direct evidence in each group to account for the overlap and methodological quality of the reviews.

**Table 6:** Taxonomy of HT systematic reviews according to key elements.

Comparisons	Systematic reviews	AMSTAR score	Review characteristics
1	Inglis et al (2010) [34]	10	Population: Stable and recently discharged patients Intervention: HT with clinical support provided during office hours or 24/7, without home visits for clinical assessment or educational purposes Comparator group: Usual care
	Clark et al (2007) [33]	7	
	Giamouzis et al (2012) [47]	2	
2	Pandor et al (2013) [37]	9	Population: Recently discharged patients (≤28 days) Intervention: HT with clinical support provided during office hours only, without home visits for clinical assessment or educational purposes Comparator group: Usual care
3	Pandor et al (2013) [37]	9	Population: Patients with stable heart failure Intervention: HT with clinical support provided during office hours only, without home visits for clinical assessment or educational purposes Comparator group: Usual care
4	Pandor et al (2013) [37]	9	Population: Patients with stable heart failure Intervention: HT with clinical support provided 24/7, without home visits for clinical assessment Comparator group: Usual care
5	Polisena et al (2010) [35]	8	Population: Stable and recently discharged patients Intervention: HT with clinical support provided during office hours or 24/7, with or without home visits for clinical assessment or educational purposes Comparator group: Usual care
	Clarke et al (2011) [36]	2	
6	Chaudhry et al (2007) [41]	5	Population: Stable and recently discharged patients Intervention: HT with clinical support provided during office hours or 24/7, with or without home visits for clinical assessment or educational purposes Comparator groups: usual care, home visits, nursing telephone support, and/or pre/post HT
	Dang et al (2009) [43]	4	
	Paré et al (2010) [45]	4	
	Martinez et al (2006) [39]	3	
	Louis et al (2003) [38]	3	
	Kraai et al (2011) [46]	2	
	Maric et al (2009) [44]	2	
	Seto (2008) [42]	2	
	Paré et al (2007) [40]	1	

### Home Telemonitoring Effects

#### All-Cause Mortality

Eleven reviews examined the effects of HT on all-cause mortality. Of them, five pooled study results into a meta-analysis [33-37]. The remaining six summarized the available evidence narratively using various qualitative or semi-quantitative techniques [38,39,41,43,45]. All reviews across the six taxonomy groups concluded that HT is effective in reducing the risk of all-cause mortality. As shown in Table 7, relative risk reductions in meta-analyses that achieved the highest AMSTAR score in each group of the taxonomy ranged from 15%, with hazard ratio (HR) 0.85 (95% credible interval [CrI] 0.59-1.2) [37] to 40%, with risk ratio (RR) 0.60 (95% CI 0.45-0.81) [35]. The absolute risk reduction (ARR) in mortality with HT ranged from 1.4% to 6.5%, respectively. Similar relative and absolute risk reductions were also observed in the other two meta-analyses that achieved lower AMSTAR scores [33,36]. The strongest evidence (moderate quality) comes from the Cochrane review that compared the effects of HT without home visits or intensified attendance at cardiology clinics with usual care. In the pooling of all-cause mortality data from 11 RCTs involving 2710 patients with HF, HT resulted in statistically significant risk reduction of 34% (RR 0.66 [95% CI 0.54-0.81]); an ARR of 5.2% (95% CI -2.9 to -7.1), equating to a number needed to treat of 19 to postpone one death. Evidence from a more recent review [37] suggests that improvements in survival rates with HT are more pronounced in patients who have been recently discharged from the hospital (HR 0.62 [95% CrI 0.42-0.89]; ARR 5% [95% CI -1.4 to -7.8]) than patients without any acute event or deterioration in the past 28 days before randomization (HR 0.85 [95% CrI 0.59-1.20]; ARR 1.4% [95% CI -3.9 to 1.9]). However, quality of evidence was low for this finding, suggesting that further research is very likely to have an important impact on this estimate.

#### All-Cause Hospitalizations

With respect to all-cause hospitalizations, most reviews reported beneficial effects with HT interventions. However, the magnitude and uncertainty of the reported estimates varied considerably across reviews due to differences in the inclusion, classification, and analysis of HT studies. Relative effects in meta-analyses that favored HT over usual care ranged from 0.99 (95% CI 0.88-1.11) [36] to 0.67 (95% CrI 0.42-0.97) [37]. The ARR in all-cause hospitalizations with HT varied from 4.7%-13.8%. As shown in Table 8, the largest relative and absolute risk reduction was seen in recently discharged patients with HF, receiving HT with clinical support during office hours. Results from two separate meta-analyses of RCTs that included patients with stable HF (ie, without any acute event or deterioration in the past 28 days prior to randomization) yielded markedly different results. HT with clinical support provided during office hours increased the risk of all-cause hospitalizations in patients with stable HF by 17% (HR 1.17 [95% CrI 0.89-1.59], with an absolute risk increase of 4.7% [95% -3.2 to 14.8]). While HT with 24/4 clinical support yielded beneficial but marginally lower relative effects than those observed in recently discharged patients: HR 0.84 (95% CrI 0.54-1.15); ARR 5.7% (-1.8 to 4.8%). Results were statistically inconclusive owing to the small number of patients and events in the included RCTs. Despite the generally favorable effects of HT observed in the reviews, quality of evidence was consistently low for the outcome of all-cause hospitalizations due to risk of bias in the primary studies and statistical heterogeneity in the meta-analyses.

#### Heart Failure Hospitalizations

A total of seven reviews examined the impact of HT interventions on HF-related hospitalizations. All of them reported beneficial effects with HT. Relative risk reductions in the four reviews that incorporated meta-analysis ranged from 14% (HR 0.86 [95% CI 0.61-1.21)] [37] to 36% (HR 0.64 [95% CrI 0.34-1.14]), while the ARR in HF-related hospitalizations extended from 3.7%-8.2%. As shown in Table 9, the strongest evidence (moderate quality) comes from a high-quality meta-analysis of 4 large RCTs (N=1570 patients), which found statistically significant risk reductions of 21% in HF-related hospitalizations with HT versus usual care, equating to an ARR of 6% (95% CI-1.7 to -9.7) [34]. Findings from a recent review [37] suggest that improvements in HF-related hospitalizations might be more pronounced in patients with stable HF receiving telemonitoring with clinical support during 24/7 (HR 0.64 [95% CrI 0.34-1.14]). HT interventions with clinical support during office hours yielded a smaller relative effect for recently discharged patients than patients with stable HF (HR 0.86 [95% CrI 0.61-1.21] versus HR 0.70 [95% CrI 0.34-1.50]). However, results were statistically inconclusive and the overall quality of evidence was found to be low, suggesting that these estimates should be interpreted with caution until more evidence accumulates.

**Table 7:** Summary of findings from the meta-analyses with the most direct evidence in each group for the outcome of all-cause mortality.^a-g^

Outcome: Comparison		Number of participants (studies), Follow-up	Quality of evidence, GRADE^a-d^	Relative effect (95% CI)	Anticipated absolute effects	
					Risk with comparator	Risk difference with HT (95% CI)
**All-cause mortality: Comparison 1 [34]**						
	Population: Stable and recently discharged patients	2710 (11 studies) 3-8 months	MODERATE due to risk of bias^e^	RR 0.66 (0.54 to 0.81)	154 per 1000	52 fewer per 1000 (from 29 fewer to 71 fewer)
	Intervention: Home telemonitoring with clinical support provided during office hours or 24/7, without home visits for clinical assessment or educational purposes					
	Comparator group: usual care					
**All-cause mortality: Comparison 2 [37]**						
	Population: Recently discharged patients (≤28 days)	1234 (8 studies) 3-12 months	LOW due to risk of bias and imprecision ^e,f^	HR 0.62 (0.42 to 0.89)^g^	139 per 1000	50 fewer per 1000 (from 14 fewer to 78 fewer)
	Intervention: Home telemonitoring with clinical support provided during office hours, without home visits for clinical assessment or educational purposes					
	Comparator group: usual care					
**All-cause mortality: Comparison 3 [37]**						
	Population: Patients with stable heart failure	1501 (7 studies) 6-12 months	LOW due to risk of bias and imprecision^e,f^	HR 0.85 (0.59 to 1.2)^g^	99 per 1000	14 fewer per 1000 (from 39 fewer to 19 more)
	Intervention: Home telemonitoring with clinical support provided during office hours, without home visits for clinical assessment or educational purposes					
	Comparator group: usual care					
**All-cause mortality: Comparison 4 [37]**						
	Population: Patients with stable heart failure	1258 (3 studies) 12-24 months	LOW due to risk of bias and imprecision^e,f^	HR 0.85 (0.58 to 1.27)^g^	143 per 1000	20 fewer per 1000 (from 57 fewer to 35 more)
	Intervention: Home telemonitoring with clinical support provided 24/7, without home visits for clinical assessment or educational purposes					
	Comparator group: usual care					
**All-cause mortality: Comparison 5 [35]**						
	Population: Stable and recently discharged patients	1200 (5 studies) 3-12 months	MODERATE due to risk of bias^e^	RR 0.60 (0.45 to 0.81)	164 per 1000	65 fewer per 1000 (from 31 fewer to 90 fewer)
	Intervention: Home telemonitoring with clinical support provided during office hours or 24/7, with or without home visits for clinical assessment or educational purposes					
	Comparator group: usual care					

^a^High quality: Further research is very unlikely to change our confidence in the estimate of effect.

^b^Moderate quality: Further research is likely to have an important impact on our confidence in the estimate of effect and may change the estimate.

^c^Low quality: Further research is very likely to have an important impact on our confidence in the estimate of effect and is likely to change the estimate.

^d^Very low quality: We are very uncertain about the estimate.

^e^Most trials did not provide details of random sequence generation, allocation concealment, and blinding of data analysts or assessors (see Multimedia Appendix 5).

^f^The optimal information size criterion was not met by the meta-analysis (power <80%).

^g^95% credible intervals (Bayesian meta-analysis).

**Table 8:** Summary of findings from the meta-analyses with the most direct evidence in each group for the outcome of all-cause hospitalizations.^a-h^

Outcome: Comparison		Number of participants (studies) Follow-up	Quality of evidence GRADE^a-d^	Relative effect (95% CI)	Anticipated absolute effects	
					Risk with comparator	Risk difference with HT (95% CI)
**All-cause hospitalization: Comparison 1 [34]**						
	Population: Stable and recently discharged patients	2343 (8 studies) 6-12 months	LOW due to risk of bias, inconsistency, and imprecision^e-g^	RR 0.91 (0.84 to 0.99)	521 per 1000	47 fewer per 1000 (from 5 fewer to 83 fewer)
	Intervention: Home telemonitoring with clinical support provided during office hours or 24/7, without home visits for clinical assessment or educational purposes					
	Comparator group: usual care					
**All-cause hospitalization: Comparison 2 [37]**						
	Population: Recently discharged patients (≤28 days)	831 (5 studies) 6-12 months	LOW due to risk of bias, inconsistency, imprecision^e-g^	HR 0.67 (0.42 to 0.97)^h^	569 per 1000	138 fewer per 1000 (from 11 fewer to 271 fewer)
	Intervention: Home telemonitoring with clinical support provided during office hours, without home visits for clinical assessment or educational purposes					
	Comparator group: usual care					
**All-cause hospitalization: Comparison 3 [37]**						
	Population: Patients with stable heart failure	1267 (5 studies) 6-12 months	LOW due to risk of bias, imprecision^e,f^	HR 1.17 (0.89 to 1.59)^h^	357 per 1000	47 more per 1000 (from 32 fewer to 148 more)
	Intervention: Home telemonitoring with clinical support provided during office hours, without home visits for clinical assessment or educational purposes					
	Comparator group: usual care					
**All-cause hospitalization: Comparison 4 [37]**						
	Population: Patients with stable heart failure	1258 (3 studies) 12-24 months	LOW due to risk of bias, inconsistency, imprecision^e-g^	HR 0.84 (0.54 to 1.15)^h^	474 per 1000	57 fewer per 1000 (from 181 fewer to 48 more)
	Intervention: Home telemonitoring with clinical support provided 24/7, without home visits for clinical assessment or educational purposes					
	Comparator group: usual care					
**All-cause hospitalization: Comparison 5 [35]**						
	Population: Stable and recently discharged patients	787 (3 studies) 3-12 months	LOW due to risk of bias, imprecision^e,f^	RR 0.79 (0.66 to 0.94)	438 per 1000	92 fewer per 1000 (from 26 fewer to 149 fewer)
	Intervention: Home telemonitoring with clinical support provided during office hours or 24/7, with or without home visits for clinical assessment or educational purposes					
	Comparator group: usual care					

^a^High quality: Further research is very unlikely to change our confidence in the estimate of effect.

^b^Moderate quality: Further research is likely to have an important impact on our confidence in the estimate of effect and may change the estimate.

^c^Low quality: Further research is very likely to have an important impact on our confidence in the estimate of effect and is likely to change the estimate.

^d^Very low quality: We are very uncertain about the estimate.

^e^Most trials did not provide details of random sequence generation, allocation concealment, and blinding of data analysts or assessors (see Multimedia Appendix 5).

^f^The optimal information size criterion was not met by the meta-analysis (power <80%).

^g^Serious unexplained inconsistency/heterogeneity (I^2^>70%). Point estimates and confidence intervals between RCTs varied considerably in magnitude and direction.

^h^95% credible intervals (Bayesian meta-analysis).

**Table 9:** Summary of findings from the meta-analyses with the most direct evidence in each group for the outcome of HF-related hospitalizations.

Outcome: Comparison		Number of participants (studies) Follow up	Quality of evidence (GRADE)^a-d^	Relative effect (95% CI)	Anticipated absolute effects	
					Risk with comparator	Risk difference with HT (95% CI)
**HF-related hospitalizations: Comparison 1 [34]**						
	Population: Stable and recently discharged patients	1570 (4 studies) 8-12 months	MODERATE due to risk of bias^e^	RR 0.79 (0.67 to 0.94)	285 per 1000	60 fewer per 1000 (from 17 fewer to 94 fewer)
	Intervention: Home telemonitoring with clinical support provided during office hours or 24/7, without home visits for clinical assessment or educational purposes					
	Comparator group: usual care					
**HF-related hospitalizations: Comparison 2 [37]**						
	Population: Recently discharged patients (≤28 days)	755 (2 studies) 6-8 months	LOW due to risk of bias and imprecision^e,f^	HR 0.86 (0.61 to 1.21)^g^	315 per 1000	37 fewer per 1000 (from 109 fewer to 52 more)
	Intervention: Home telemonitoring with clinical support provided during office hours, without home visits for clinical assessment or educational purposes					
	Comparator group: usual care					
**HF-related hospitalizations: Comparison 3 [37]**						
	Population: Patients with stable heart failure	432 (2 studies) 12 months	LOW due to risk of bias and imprecision^e,f^	HR 0.70 (0.34 to 1.5)^g^	221 per 1000	61 fewer per 1000 (from 139 fewer to 91 more)
	Intervention: Home telemonitoring with clinical support provided during office hours, without home visits for clinical assessment or educational purposes					
	Comparator group: usual care					
**HF-related hospitalizations: Comparison 4 [37]**						
	Population: Patients with stable heart failure	1170 (3 studies) 12-24 months	LOW due to risk of bias and imprecision^e,f^	HR 0.64 (0.34 to 1.14)^g^	251 per 1000	82 fewer per 1000 (from 157 fewer to 30 more)
	Intervention: Home telemonitoring with clinical support provided 24/7, without home visits for clinical assessment or educational purposes					
	Comparator group: usual care					

^a^High quality: Further research is very unlikely to change our confidence in the estimate of effect.

^b^Moderate quality: Further research is likely to have an important impact on our confidence in the estimate of effect and may change the estimate.

^c^Low quality: Further research is very likely to have an important impact on our confidence in the estimate of effect and is likely to change the estimate.

^d^Very low quality: We are very uncertain about the estimate.

^e^Most trials did not provide details of random sequence generation, allocation concealment, and blinding of data analysts or assessors (see Multimedia Appendix 5).

^f^The optimal information size criterion was not met by the meta-analysis (power <80%).

^g^95% credible intervals (Bayesian meta-analysis).

#### Cost Savings

Eleven reviews examined the effects of HT interventions on cost savings, but none of them pooled results into a meta-analysis due to the inconsistency in cost-analysis methods used in the original studies. One review of both RCTs and observational studies that focused explicitly on cost savings as an outcome found direct cost reductions to the health care system from HT compared to usual care, which ranged between 1.6% and 68.3% [42]. Eight reviews concurred that the impact of HT interventions on health care costs appeared to be positive in more cases than not, but in general results were statistically inconclusive and varied depending on the context and specific national health system of the study [34,36,38,39,41,43,44,47]. Identified cost reductions, taken individually and collectively, were mainly associated with savings from reduced expenditures on hospitalizations and, to a lesser extent, from home visits and patient travel costs. Although none of the systematic reviews used standardized instruments or validated methods to formally appraise the quality of economic evaluations in the original studies, most reviews criticized the methodologies adopted in these studies and strongly recommended that future research rigorously conduct cost-effectiveness assessments of HT in adequately powered RCTs.

#### Quality of Life

Eight reviews included health-related quality of life as an outcome measure. All of them summarized the available evidence qualitatively owing to the different assessment instruments used in the primary studies. Overall, reviews concluded that HT improved quality of life. However, such inferences were not supported by the study-level evidence they presented. For example, in one of the high-quality reviews, the authors concluded that HT improves quality of life, but only three of the seven studies that reported data for this outcome found positive and statistically significant improvements. In most reviews, data extraction and reporting pertaining to this outcome was inadequate, since authors focused on the statistical significance of study results rather than the direction and magnitude of effect [33-35,39,44].

#### Length of Stay

The impact of HT on hospital length of stay due to exacerbated HF events and/or any cause hospitalization was examined in eight reviews. Results for this outcome were tabulated and summarized narratively. Three reviews, all of which focused on RCTs, concluded that the impact of HT on length of stay was ambivalent [34,36,37]. However, the remaining systematic reviews reached different conclusions [35,39,40,43,45]. Reviews that incorporated observational studies in their analysis and a broader set of interventional studies with various comparator groups (eg, home visits and nurse telephone support) concluded that HT reduces length of stay [39,40,45].

### Opening the Black Box of Home Telemonitoring Technologies

#### Description

As described earlier, the extent to which various technological devices may have an impact on the effectiveness of HT has not been investigated systematically in previous reviews. Despite the different forms and generations of HT that have emerged over the years as a result of the continuous technological advances and efforts to improve remote monitoring of patients with HF, most systematic reviews have treated HT as a black box, paying little or no attention to the technology component.

To investigate this issue further, we extracted data from the primary studies included in the 15 reviews and conducted a series of post-hoc analyses. Our main goal was to gain further insights into the various types of HT technologies in use and investigate the link between HT technologies and clinical effectiveness. Put simply, we explored the following question: Does HT technology matter? Our results are presented in the following two subsections.

#### Toward a Taxonomy of Home Telemonitoring Interventions

##### Data Extraction and Post-Hoc Analyses

**Figure 2:**
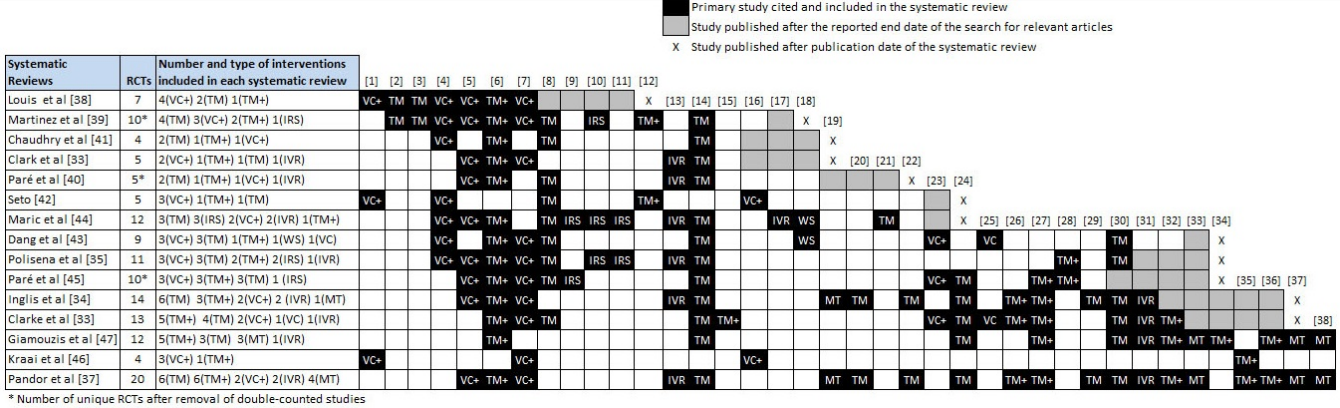
Citation matrix of previously published RCTs included in the 15 systematic reviews (all references are available in Multimedia Appendix 3).

**Figure 3:**
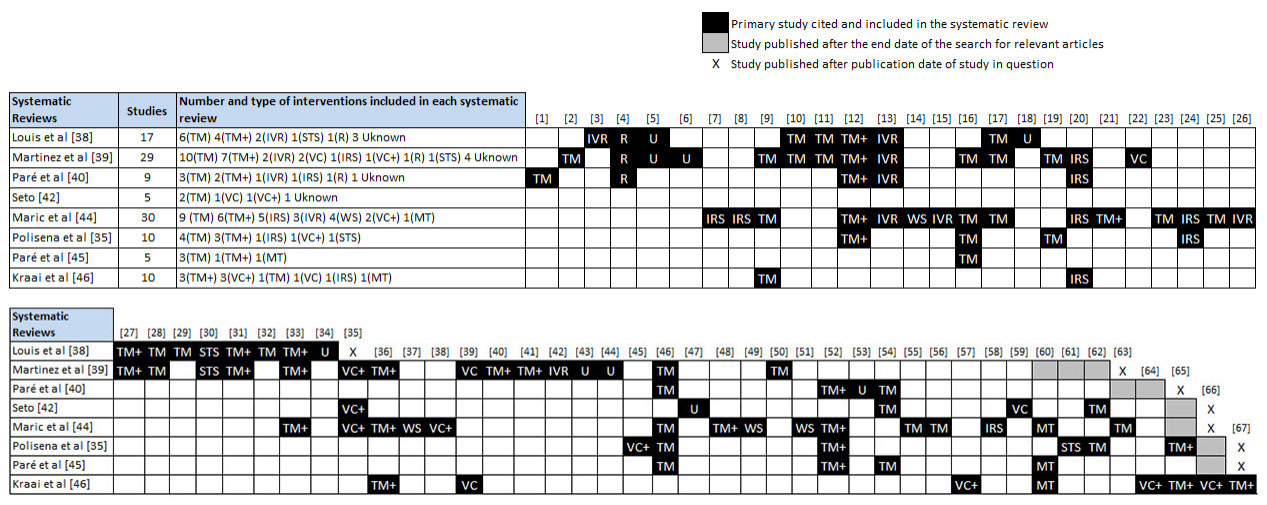
Citation matrix of previously published observational studies included in the 15 systematic reviews (all references are available in Multimedia Appendix 3).

Building on the citation matrices presented earlier (Multimedia Appendix 3), we retrieved all primary studies included across the 15 reviews, extracted relevant information from each study about the different technologies and monitoring approaches in use, and subsequently classified HT interventions into groups according to the technology in use (Figures 2 and 3). We were able to extract data from all 105 primary studies, with the exception of 8 observational studies that could not be retrieved. These are denoted in Figure 3 with the letter “U” (Unknown). By carefully reading through the detailed descriptions of the interventions provided in the primary studies, we identified five main types of HT interventions as follows.

##### Video Consultation

Seven RCTs and 9 observational studies across 14 systematic reviews involved real-time, two-way synchronous communication between patients and caregivers via the use of specialized videoconferencing equipment. In most of these studies (6 RCTs, 6 observational), which are denoted with the acronym “VC+”, patients received scheduled video-consultations using specialized videoconferencing equipment and peripheral devices that were either interconnected with the main videoconferencing unit or were capable of independently transmitting basic physiological measures through telephone (trans-telephonic transmission) or broadband connection. In the studies that used videoconferencing equipment with integrated electronic stethoscopes and blood pressure monitors, nurses used a headset at the receiving station to hear cardiac sounds and video-consult with patients about items such as weight, symptoms, and observance. This process allowed in-depth assessment and triage at pre-scheduled times (eg, daily or twice weekly) without patients having to leave home. In the remaining studies (1 RCT and 3 observational), the intervention involved use of stand-alone videoconferencing equipment without transmission of vital signs. These studies are denoted as “VC”.

##### Automated Device-Based Telemonitoring

A total of 23 RCTs and 37 observational studies across 15 systematic reviews utilized various non-invasive electronic devices, to support remote patient monitoring and automated transmission of vital signs and/or symptoms. These interventions were further categorized into three mutually exclusive subgroups, based on the technological characteristics of the intervention, as well as methods of data collection and transmission.

The first subgroup of studies (10 RCTs and 20 observational) employed patient-initiated electronic devices, such as wired or wireless weight scales and blood pressure monitors, capable of measuring and automatically transmitting data from the patient at home to a Web-based server through dedicated telecommunication stations (eg, modems or broadband connection boxes). These studies are denoted as “TM”. The second subgroup (10 RCTs and 12 observational studies) also involved the use of patient-initiated electronic devices for monitoring and transmission of vital signs, but contrary to the first subgroup, these devices were interconnected with individualized symptom response devices that prompted patients about a heart-healthy diet, physical activity, and medication compliance, and requested answers (Yes/No) to relevant questions about HF-related symptoms. Patient responses along with monitored vital signs were recorded and automatically uploaded to a central server through a telephone line or broadband connection. Clinicians were able to monitor the patients, provide advice, and update treatment regimens by accessing data via standard browser interfaces. Studies belonging to this subgroup are denoted as “TM+”. The third subgroup of studies (3 RCTs and 5 observational) cited across 6 systematic reviews involved the use of stand-alone, interactive symptom response devices without capability of transmission of physiological measures or vital signs. Studies in this subgroup are denoted as “IRS”.

##### Web-Based Telemonitoring

One RCT and 4 observational studies cited across 2 systematic reviews examined the effectiveness of secured websites for the purpose of patient remote monitoring. Interventions involved the use of personal computers or laptops with Internet access, allowing patients gain secure access to a Web-based portal to manually enter their vital signs obtained through stand-alone peripheral devices (eg, blood pressure monitors, weight scales, sphygmomanometer, and pedometer), answer a range of questions about symptoms, and receive feedback, as well as educational material. Web-based telemonitoring studies are denoted with the acronym “WS”.

##### Interactive Voice Response Systems

A total of 3 RCTs and 5 observational studies cited across 9 systematic reviews utilized automated interactive voice response systems (similar to the ones used by airline companies and banks) that required manual input of data by the patient using the telephone keypad of their home or mobile telephone to send information concerning vital signs and symptoms to health care providers. Each parameter was entered by the patient in reply to a computer-generated question asked by a recorded voice. The system generated alerts to the study nurse when pre-specified symptoms or physiologic changes were detected. Studies that implemented an automated interactive voice response system are denoted as “IVR”.

##### Mobile Telemonitoring

Four RCTs and 1 observational study across 6 systematic reviews (denoted in Figures 2 and 3 as “MT”) involved telemonitoring of patients with HF via the use of mobile devices such as mobile phones and personal digital assistants (PDAs). In 3 RCTs, patients were instructed to use on a daily basis external electronic devices that were interconnected via Bluetooth to their mobile phone or PDA allowing measurement and automatic forwarding of physiological parameters and vital signs (eg, electrocardiogram, blood pressure, weight) to a central server. In the other two studies, the process of measurement and transmission was not automated. After establishing an Internet connection using the micro browser of their mobile device, patients had to manually enter their vital signs and physiological parameters using data-entry templates generated in Wireless Markup Language (WML).

Three observational studies cited across 4 systematic reviews involved the use of other communication platforms that could not be classified under any of the above categories. Two used structured telephone support only (denoted in Figure 3 as “STS”), while a third study used a pager to transmit computer-generated reminders to the patient to take medications, weigh themselves, and measure their blood pressure and heart rate. Patients were then contacted by phone once a week to verbally communicate to the nurse their physiological data. This study is denoted in Figure 3 as “R” (Reminders).

#### Exploratory Analysis of the Effectiveness of Home Telemonitoring Technologies

##### Description

Having identified the main types of HT interventions included in the systematic reviews, we now turn our attention to the question of whether technology has an impact on the effectiveness of HT and whether it can explain variations in the direction and magnitude of the observed effect sizes with respect to all-cause mortality, all-cause hospitalizations, and HF-related hospitalizations.

Using data from the three systematic reviews in our sample with the highest quality assessment scores in Table 6 [34,35,37], we conducted a series of exploratory subgroup meta-analyses for each type of HT intervention that had at least 2 studies. More specifically, we identified all RCTs of both recently discharged and stable HF patients included in the systematic reviews (Figure 2), extracted all relevant data by outcome (ie, number of events and patients in each group), cross-checked and validated all data for accuracy, and then grouped these studies according to the specific type or category of HT technology used. All subgroup analyses were restricted to RCTs that compared HT without home visits to usual care. Meta-analyses were performed using risk ratios, intention-to-treat analysis, the Mantel-Haenszel statistical method, and a random-effects analysis model to account for functional differences between interventions.

Four categories of HT interventions were identified among the 20 RCTs that were cross-referenced in the three systematic reviews. These included (1) automated device-based telemonitoring (TM and TM+), (2) mobile telemonitoring (MT), (3) automated interactive voice response (IVR), and (4) video-consultation with trans-telephonic vital signs monitoring (VC+). Table 10 presents the summary effect size for each type of HT intervention. Forest plots for each meta-analysis are presented in Multimedia Appendix 6. Wherever a meta-analysis could not be carried out due to insufficient number of studies, we present the results of the single trials instead.

##### Automated Device-Based Telemonitoring

Meta-analysis of 12 RCTs comparing device-based telemonitoring with usual care showed a statistically significant relative reduction of 35% in all-cause mortality (RR 0.65 [0.54-0.79], *P*<.001). Similarly, the relative risk of HF-related hospitalizations was reduced by 23% (RR 0.77 [0.64-0.91], *P*=.003). However, the number of studies reporting this outcome was significantly smaller (5 RCTs). The impact of automated device-based telemonitoring on all-cause hospitalization was also positive but statistically inconclusive (RR 0.89 [0.76-1.05], *P*=.17). Studies that involved telemonitoring of vital signs and symptoms through individualized symptom response systems (TM+) yielded slightly smaller effects with respect to all-cause mortality and all-cause hospitalizations than studies involving telemonitoring of vital signs only (TM). However, this difference was not statistically significant.

##### Mobile Telemonitoring

The effectiveness of mobile telemonitoring versus usual care was examined in 4 RCTs. All studies showed a beneficial trend in reducing all-cause mortality (RR 0.67 [0.35-1.26], *P*=.21). Similarly, the relative risk of HF-related hospitalizations was reduced with mobile telemonitoring by 28% (RR 0.72 [0.42-1.26], *P*=.25). However, results for both of these outcomes were statistically inconclusive owing to the small number and size of RCTs in this group.

##### Interactive Voice Response

The effectiveness of IVR was examined in only two RCTs. Contrary to other types of HT interventions, use of IVR systems was not associated with reductions in the relative risk of all-cause mortality, all-cause hospitalizations, and HF hospitalizations in either of these trials.

**Table 10:** Effects of HT according to the type of technology used.

Types of HT technologies	All-cause mortality	All-cause hospitalizations	HF hospitalizations
Automated device-based telemonitoring (TM & TM+)	RR 0.65 [0.54-0.79], *P*<.001, I^2^=0% (12 RCT)	RR 0.89 [0.76-1.05], *P*=.17, I^2^=66% (10 RCT)	RR 0.77 [0.64-0.91], *P*=.003, I^2^=25% (5 RCT)
Telemonitoring of vital signs (TM)	RR 0.64 [0.51-0.80], *P*<.001, I^2^=0% (6 RCT)	RR 0.81 [0.64-1.03], *P*=.08, I^2^=76% (6 RCT)	RR 0.73 [0.58-0.91], *P*=.005, I^2^=32% (4 RCT)
Telemonitoring of vital signs and symptoms (TM+)	RR 0.70 [0.47-1.04], *P*=.08, I^2^=12% (6 RCT)	RR 1.04 [0.90-1.21], *P*=.58, I^2^=0% (4 RCT)	RR 0.87 [0.66-1.13], *P*=.29^a^
Mobile telemonitoring (MT)	RR 0.67 [0.35-1.26], *P*=.21, I^2^=44% (4 RCT)	RR 0.99 [0.76-1.29], *P*=.94, I^2^=54% (3 RCT)	RR 0.72 [0.42-1.26], *P*=.25, I^2^=48% (2 RCT)
Interactive voice response (IVR)	RR 1.09 [0.57-2.07], *P*=.80 I^2^=0% (2 RCT)	RR 1.18 [0.87-1.60], *P*=.29^a^	RR 1.03 [0.65-1.61], *P*=.91^a^
Video-consultation with vital signs monitoring (VC+)	RR 0.95 [0.35-2.53], *P*=.91, I^2^=0% (2 RCT)	RR 1.06 [0.97-1.16], *P*=.22^a^	No studies available
All types of HT combined (TM, TM+, MT, IVR, VC+	RR 0.73 [0.62-0.85], *P*<.001, I^2^=0% (20 RCT)	RR 0.95 [0.85-1.06], *P*=.38, I^2^=66% (15 RCT)	RR 0.79 [0.69-0.91], *P*=.001, I^2^=19% (RCT=8)

^a^Meta-analysis could not be performed. Only 1 RCT provided data.

##### Videoconferencing With Vital Signs Monitoring

Only two RCTs investigated the effectiveness of videoconferencing with trans-telephonic monitoring of vital signs. No significant differences were found for all-cause mortality (RR 0.95 [0.35-2.53], *P*=.91) owing to the small number of patients and events. Neither of these studies reported results for the outcome of HF-related hospitalizations.

##### All Types of Home Telemonitoring Interventions Combined

As a final step, we pooled data from all the RCTs in an effort to further explore the effectiveness of HT, by combining the findings of the systematic reviews with the highest methodological quality to increase statistical power and precision. As shown at the bottom of Table 10, when considered collectively, HT interventions without home visits are associated with a statistically significant, relative risk reduction of all-cause mortality (RR 0.73 [0.62-0.85], *P*<.001) and HF-related hospitalizations (RR 0.79 [0.69-0.91], *P*=.001) of 27% and 21%, respectively. However, there was no significant reduction in the relative risk of all-cause hospitalizations (RR 0.95 [0.85-1.06], *P*=.38). Results pertaining to all-cause hospitalizations were also associated with high statistical heterogeneity (I^2^ 66%) due to differences in both direction and magnitude of effects between the included studies.

## Discussion

### Summary of the Evidence

This overview appraised and summarized evidence from 15 systematic reviews assessing the effects of HT interventions on patients with HF. To our knowledge, it is the first synthesis of systematic reviews to take a broad perspective on evidence-based telemonitoring in HF. It is also the first overview to investigate the nature of the link between different types of HT technologies and outcomes.

The systematic reviews included in our evidence synthesis covered a broad family of complex HT interventions rather than a standardized type of HT, involving various technologies and monitoring approaches that were supplemented with various other components in the context of comprehensive care programs (eg, clinical advice via telephone, patient education, and in some cases home visits). Despite ostensibly being reviews of the same body of literature as their research objectives suggest, we identified several key differences between them with respect to the scope of inquiry, study selection criteria, classification, and analysis of HT studies.

To best organize and synthesize the evidence, we performed a citation analysis and developed a taxonomic structure to categorize the included reviews into homogeneous groups according to their common elements and PICO characteristics. Subsequently, we appraised the methodological quality of the reviews and constructed summary of findings tables to present the effects of HT interventions from the most direct evidence, that is, from reviews that achieved the highest methodological quality score in each classification group. Limitations in the quality of evidence were formally reflected in the summary of findings tables by outcome, using the evidence grading system developed by the GRADE group [17] and in the analysis by interpreting results and formulating statements about the effectiveness of HT in light of the risk of bias in the primary studies. We also conducted a series of post-hoc analyses to develop a preliminary taxonomy of HT technologies and then investigate the link between these technologies and HT effectiveness.

Looking both collectively and individually across the included systematic reviews, this overview demonstrates that there is no high-quality evidence for or against the effectiveness of HT interventions for HF patients. There is moderate quality evidence that HT interventions with clinical support provided during office hours or 24/7 reduce the risk of all-cause mortality and HF-related hospitalizations compared to usual care. Yet the bulk of the literature consists of low-quality and inconsistent evidence about the beneficial effects of HT on all-cause hospitalizations [34,37]. Risk reductions in mortality and all-cause hospitalizations appear to be greater in patients who have been recently discharged from an acute care setting after an HF exacerbation and are at high risk of re-hospitalization or sudden death, while improvements in HF-related hospitalizations appear to be more pronounced with 24/7 HT on patients with stable HF. However, these results should be interpreted with caution and be considered as hypothesis-generating in future trials and systematic reviews, given the large uncertainty (imprecision) in the estimates of effect. Evidence about cost-effectiveness remains limited, and there are no reliable data on the long-term benefits and economic implications of HT interventions [51]. Despite current indications in the literature that HT can generate cost savings for health care providers and national health care systems [42], the economic evidence base is still weak and fails to meet generally accepted standards of economic analysis. With respect to the effects of HT on hospital length of stay and quality of life, there is no consistent evidence from which to draw robust conclusions.

The results of the exploratory post-hoc analyses we conducted show that the majority of interventions included in the 15 systematic reviews (62% of RCTs and 55% of observational studies) involved the use of non-invasive, patient-initiated electronic devices and/or interactive response systems capable of measuring and automatically transmitting vital signs, physiological data, and/or symptoms from the patient at home to the health care professionals providing care and clinical feedback. Other less frequent types of HT interventions included in the systematic reviews involved the use of video-consultation equipment (18% of RCTs and 13% of observational studies), mobile telemonitoring through mobile phones and PDAs (10% of RCTs), and automated interactive voice response systems requiring manual data entry by the patient (8% of RCTs and 7% of observational studies). Therefore, it can be argued that the results of the systematic reviews included in this overview reflect for the most part the effectiveness of “automated device-based HT interventions” and, to a lesser extent, the effects of interventions involving other technologies and monitoring approaches. In fact, the effects of the other types of HT technologies identified in our analysis (eg, videoconferencing, mobile telemonitoring and interactive voice response) have largely been masked in prior systematic reviews due to the fact that virtually all of them have treated HT as a “single-type intervention”. This is further supported by the results of the exploratory meta-analyses we conducted, which show that not all types of HT technologies are equally effective. Yet, when pooled together into one large group of HT interventions, the category with the most trials that has the largest impact on the results (ie, automated device-based telemonitoring) masks valuable insights about the effects of the other interventions. For example, mobile telemonitoring, which has emerged as a relatively new approach due to the ubiquitous nature of mobile devices and cell phones despite the small number of available studies, is associated with beneficial trends showing promise in reducing mortality and HF-related hospitalizations. None of the prior systematic reviews included in our study identified or commented on this. On the other hand, interventions using interactive voice response systems and video-consultations were not associated with beneficial effects on all-cause mortality and hospitalizations. However, when trials from these distinctively different interventions were pooled together with the dominant group of studies (automated device-based telemonitoring), the relative risk reduction of all-cause mortality and HF-related hospitalizations remained statistically significant. This indicates that the “one size fits all” approach that has been used so far in prior systematic reviews and meta-analyses in the field of HT may not be appropriate.

### Systematic Reviews Published Since Completion of the Main Search

On December 3, 2014, we re-ran our search strategy to identify new systematic reviews published after our main search was completed. We identified 3 reviews [52-54], two of which contained meta-analysis for at least one outcome of interest. One review examined the effects of HT interventions on patients with HF and conducted several subgroup meta-analyses of RCTs containing 40 or more patients to determine which HT model is more effective and for which patient population [52]. The other two reviews assessed the effectiveness of several other “disease management” and “transitional care interventions” (eg, structured telephone support, home visiting programs, cognitive training, and invasive telemonitoring interventions) in addition to non-invasive HT. Outcomes were analyzed and reported separately for each intervention and hence, both reviews were deemed eligible for inclusion. The methodological quality of the two reviews [52,54] was found to be low (AMSTAR=2), contrary to the third review [53], which was conducted for the Agency for Healthcare Research and Quality [55] and met most of the AMSTAR criteria achieving a score of 9. All-cause and HF-related hospitalizations were reported in 2 of the 3 reviews [53,54], while findings pertaining to all-cause mortality were reported in all three.

The systematic review with the highest AMSTAR score, contrary to the other reviews included in our main analysis, concluded that HT interventions are not effective in reducing the overall risk of all-cause mortality, all-cause hospitalizations, and HF-related hospitalizations over a period of 6 months compared to usual care: *all-cause mortality at 3-6 months*: RR 0.93 (95% CI 0.25-3.48) 3 RCTs, 564 patients, “low quality of evidence”; *all-cause hospitalizations at 30 days*: RR 1.02 (95% CI 0.64-1.63) 1 RCT, 168 patients, “insufficient quality of evidence”; *all-cause hospitalizations at 3-6 months*: RR 1.11 (95% CI 0.87-1.42) 3 RCTs, 434 patients, “moderate quality of evidence”; and *HF-related hospitalizations at 3-6 months*: RR 1.70 (95% CI 0.82-3.51) 1 RCT, 182 patients, “moderate quality of evidence” [53,55]. However, this review differed in scope from all previously published systematic reviews in that it included only RCTs of adult patients recruited during or within only 1 week of an index hospitalization for HF. Also, the required timing of outcome measurement had to occur no more than 6 months from the index hospitalizations in order for RCTs to be eligible for inclusion. The use of such narrow scope and eligibility criteria limits the applicability and external validity (generalizability) of this review, the results of which should be interpreted with caution as they rely on a very small number of RCTs (≤3 per outcome), insufficient for drawing meaningful conclusions about the effectiveness of HT interventions on recently discharged (≤7 days) patients with HF.

In the second systematic review that contained meta-analysis, Nakamura et al [52] sought to investigate which HT model is more effective in reducing all-cause mortality in patients with HF. In this line of thought, they conducted a series of subgroup analyses across 13 RCTs (3337 patients) by age, severity of illness, measurement frequency, medication management, and speed of intervention. According to the findings of this review, studies in which clinical intervention was performed within one day of a change in the patient’s vital signs (termed by the authors as “rapid intervention”) had statistically significantly lower mortality rates compared to the group of studies in which clinical intervention took place later (RR 0.59 vs 0.88, *P*=.05). Also, the risk for all-cause mortality was found to be lower in HT studies that (1) had high measurement frequency of vital signs (more than twice a week vs ≤ once a week: RR 0.62 vs 0.89, *P*=.07), (2) included patients with a mean age of 65 years or over (RR 0.63 vs 0.71, *P*=.60), (3) had 70% or more of patients classified under NYHA class III or IV (RR 0.63 vs 0.86, *P*=.13), and (4) included a medication management component (RR 0.65 vs 0.85, *P*=.19) [52]. However, it is important to note that these findings are observational in nature and suffer from important limitations [56,57], including possible bias introduced through confounding by other study-level characteristics; misclassification of certain RCTs providing insufficient or no information at all for some categories; and arbitrary selection of cut-off points without any supporting evidence from sources other than the included RCTs, suggesting possible data dredging. Indeed, it is difficult to explain or justify the authors’ motivation for the selection of the cut-off points used to classify studies into subgroups. Also, it is not possible to discern which of the investigated characteristics explain, and to what extent, the observed differences in the magnitude of effects (quantitative interaction) between the included RCTs, when several studies involving frequent measurement of vital signs and “rapid intervention” by clinicians, also included older patients with more severe HF (stages III and IV). In light of these and many more limitations associated with the nature of these observational investigations, the findings of this systematic review should be interpreted with extreme caution and at best be considered as hypothesis-generating rather than hypothesis testing.

The third systematic review identified by our recent search included 14 RCTs (5021 patients), within the broader scope of HF disease management programs, evaluating the efficacy of non-invasive HT support [54]. Using vote counting by statistical significance as the main method of analysis, the authors of this review found that only 2 RCTs demonstrated a significantly positive effect on all-cause mortality, and only 3 RCTs significantly reduced all-cause and HF-related hospitalizations. Therefore, it was concluded that current evidence supporting the efficacy of HF disease management programs (including non-invasive HT interventions) demonstrates highly inconsistent results, and therefore one approach applied to a broad spectrum of different patient types may not be effective. However, it should be borne in mind that vote counting by statistical significance is inadequate to answering the question of whether there is any evidence of an effect [56]. Furthermore, vote counting has a notorious record for being misleading (p. 252 [58]), as in the case of this review where many of the included RCTs were not sufficiently powered to reach statistically significant results, leading the authors to the perception that these studies yielded “conflicting results”, although the treatment effects in these RCTs were actually similar or even larger than the ones in the studies that were statistically significant.

Our search also identified 2 recent publications that conducted post-hoc subgroup analysis of the results contained in the Cochrane systematic review included in our main results. The main objectives of these studies was to determine whether age is a factor in the success or failure of remote monitoring interventions in HF (including HT) [59] and the extent to which technological differences have an impact on the primary outcomes of interest [60]. Similarly to this overview, the study by Conway et al [60] underscores the important need to characterize HT interventions according to the technology component in use and to investigate the link between technology and HT effectiveness. However, in contrast to Conway et al [60], this overview relied on 15 systematic reviews and 105 studies to derive a preliminary taxonomy of HT technologies. Therefore it provided a richer and more comprehensive classification of telemonitoring technologies and incorporated twice as many RCTs in the meta-analysis. Briefly, the main inferences that were extracted from the two studies identified are as follows. Older people (≥70 years) with heart failure seem to benefit from HT interventions (all-cause mortality: RR 0.56 [95% CI 0.41-0.76] 4 RCTs; all-cause hospitalizations: RR 0.89 [95% CI 0.80-1.00] 3 RCTs), despite a popularly held belief of the opposite among clinicians [59]. Given the observational nature of this analysis, however, the authors stated that “discrimination by age alone may not be appropriate when inviting participation in a remote monitoring service for HF” [59]. Furthermore, evidence of systematic bias identified in the body of literature towards recruitment of individuals younger than the epidemiological average constitutes a significant problem that should be addressed in future RCTs, given the fact that HF becomes more prevalent as age is increased [59]. With respect to the impact of technological differences, the authors found that unlike other (broadly defined) HT technologies, interactive voice response systems requiring manual data entry by the patients may not be effective in reducing mortality and hospitalizations [60]. However, the number of studies included in the subgroup analysis was insufficient to draw definitive conclusions. Therefore, incorporation of new evidence in systematic reviews from recent RCTs is expected to provide further insights. This finding is consistent with the results of our post-hoc meta-analysis. Finally, Conway et al [60] argue that consideration should be given to measuring more than weight in telemonitoring interventions, as change in weight may not be sensitive enough to detect worsening of HF.

### Overall Completeness and Applicability of the Evidence

While reductions in mortality and HF-related hospitalizations found in the systematic reviews included in our main analysis are particularly encouraging and HT as a research area has witnessed considerable growth over the years expanding its evidence base, there still remain important uncertainties around the general applicability and long-term efficacy of HT interventions due to several gaps and methodological weaknesses in prior research. First, most outcome data included in the systematic reviews are drawn from interventional studies that are clinically heterogeneous in terms of duration of follow-up, measures transmitted to the care providers (eg, weight, blood pressure, symptoms, and electrocardiogram), types of HT modalities used, frequency of data transmission, as well as diagnostic criteria used for the selection of patients with HF [37]. Second, the definition of usual care and the health services provided to patients in the control group also differed between primary studies in terms of intensity, clinical visits, patient education/training, or telephone support calls, depending on the country, area, and health care organization where the study was conducted and the model of care that was implemented [36]. Third, primary studies included in the systematic reviews were performed at different intervals over an extended period of time (12 years), during which both usual care and HT technologies have markedly evolved, witnessing important improvements. The impact of these temporal changes on the treatment effects, as well as the age and clinical/pragmatic differences between the primary studies may have been an important confounding factor in the observed results. As Gurne et al [61] note, some of the very first studies of HT included in the systematic reviews were conducted in the late 1990s (see Multimedia Appendix 3), when beta-blockers were not used as consistently as they are today in patients with HF. Also, the delivery of usual care for HF has improved over the last 15 years in many developed countries with the progressive introduction of multidisciplinary care, patient education, counseling services, home visits, and self-management programs led by specialist nurses—all of which have been shown to reduce mortality and hospitalizations [62]. The extent to which improvements in the conventional methods for delivering care may have minimized the gap between HT and standard care remains unclear. One of the frequently discussed challenges in the reviews was that in most primary studies the control group was not clearly described, compromising the reviewers’ ability to understand the context the study was conducted in and how it might translate to other settings.

When interpreting the effects of HT interventions, besides the different types of technologies, it is also important to consider the technological advances that have occurred over the years (eg, in analytics, user-interfaces, and devices) and the different generations of HT technologies that have been developed. The sophistication of the technology, aside from changes in the models of care, is likely to have played an important role in outcomes. For example, as Anker et al maintain [8], first-generation HT systems, used in some of the early trials included in the reviews, were mainly “non-reactive data collection and analysis systems” that connected to external devices (eg, blood pressure and pulse monitors) utilizing conventional telephone lines to transfer physiological measures from the patient’s home to a central server accessed by clinicians. Data transfer was generally asynchronous and the care providers could not respond instantaneously. Furthermore, these systems did not provide any patient advice, education, or automated feedback. Second-generation HT systems were more interactive from a patient perspective. They used approximately the same assessment measures (weight, heart rate, blood pressure, etc) but utilized patient medication reminders, educational components, as well as feedback mechanisms. They also involved additional and more sophisticated sensors for real-time transmission of vital signs and symptoms to the care providers [8]. Although delays in detection of patient deterioration and clinical intervention could potentially occur in cases where the systems were active only during office hours, it is likely that their impact on patient outcomes was more direct than that of first-generation systems [8]. Third-generation and fourth-generation HT technologies, which provide constant analytical and decision-making structures involving mobile phones, new sensors, as well as invasive and non-invasive devices that can measure heart, lung, and/or fluid retention more accurately, might deliver even greater health gains than HT systems of previous generations [63]. Despite recent attempts by researchers (eg, [37,44]), including attempts of this overview, to separate the effectiveness of different HT interventions and modalities and identify the type of patient population that benefits the most, there is still a lack of sufficient and high-quality studies to clearly indicate which types of HT technologies and strategies provide optimum clinical benefit, under what circumstances, and for which patient subgroup. The duration for which HT would continue to confer benefits also remains unclear. A frequently cited challenge, which we encountered too during the post-hoc analysis, is that most primary studies do not provide sufficient contextual information about the intervention and control group(s). Furthermore, results are presented in a manner that does not allow stratification of the benefits across strategies, stages of illness, and patient population [34,37]. Consequently, uncertainties remain around the determinants of successful HT programs. Subgroup differences ideally require individual-level data, and meta-analyses of individual-level data simply do not exist in the field of HT. An additional inhibiting factor that has been cited by several researchers [7,9,64] includes the lack of a commonly accepted taxonomy for classifying HT interventions into meaningful groups according to the technology in use and other key characteristics (eg, intensity and complexity, health care professionals involved in the delivery of clinical feedback, and response time). It is our hope that the taxonomy of HT technologies provided in this overview will serve as a valuable resource and also as a starting point for those that conduct systematic reviews and clinical trials in the area of HT. We strongly encourage researchers who start a systematic review to build on our classification scheme to explore the extent to which differences in the technologies used by HF patients have an impact on HT outcomes.

### Quality of Evidence

In this overview, we identified and formally reflected in the summary of findings tables a number of serious limitations that we encountered during the appraisal of the primary studies and meta-analyses, which subsequently led us to rate down our confidence (quality of evidence) in the estimates of effect by outcome, following the methodological guidelines suggested by the GRADE group [17-27].

First, a high proportion of RCTs (>50%) included in the systematic reviews we examined did not provide sufficient details about random sequence generation, allocation concealment, attrition, and blinding of data collectors or outcome assessors, while in several studies there were significant differences in the baseline comparability of important prognostic factors [34,37]. As well, one third of the trials contributing data to the primary outcomes of interest received commercial funding from HT solution providers [37]. Receipt of such funding has been shown in other scientific fields to systematically bias the results in favor of the products made by the companies that fund the research [65]. Overall, as shown in Multimedia Appendix 5, most trials included in the reviews we examined contained important limitations in the design and/or execution. Therefore, quality of evidence was rated down by one level (from high to moderate) in all primary outcomes to reflect that most of the relevant evidence about the effectiveness of HT comes from studies with high or unclear risk of bias.

Second, given that the evidence base consists mainly of small trials that usually are not adequately powered to detect meaningful differences in outcomes, several meta-analyses included in this overview (eg, Comparisons 2, 3, and 4) did not meet the optimal information size criterion [22] required to establish a high level of confidence and therefore, lacked precision. The 95% credible intervals of the pooled effect crossed the line of “no effect” (1.0) and included appreciable benefit (HR<0.75) or harm (>1.25), or even both, suggesting that the effectiveness of HT in a randomly chosen study can vary substantially if the upper versus the lower boundary of the credible intervals represented the truth. Owing to the large uncertainty in the pooled estimates of effect, quality of evidence was rated down for imprecision [22].

Third, a high degree of statistical heterogeneity (eg, I^2^>50%) in study results pertaining to all-cause hospitalizations was reported in many meta-analyses. Some trials included in the reviews found HT to be associated with substantially beneficial effects (RR 0.36), while others showed that HT increased the relative risk of all-cause hospitalizations versus usual care (RR 1.18). However, none of the reviews was able to identify potential effect modifiers that might explain the observed heterogeneity.

In short, risk of bias in the primary studies coupled with large and unexplained inconsistencies or imprecision in meta-analyses, inevitably decrease one’s confidence (quality of evidence) in the estimates of HT effects.

### Potential Biases in the Overview Process

This overview adopted and applied rigorous methods suggested by the Cochrane Collaboration [11] with a view to minimizing the impact of bias arising from different sources within and across systematic reviews, as well as the overview process itself. Strengths of our approach include the use of sensitive and comprehensive search methods to identify all relevant reviews, the duplicated process applied in study selection, data extraction, and methodological quality appraisal, as well as the use of the GRADE system to rate the quality of evidence for each primary outcome.

Nonetheless, overviews are inevitably constrained by the quality and reporting characteristics of the systematic reviews, the quality of evidence within reviews, and the time lag between the publication of original studies and the reviews. Taking published systematic reviews as the sole evidence source and not searching for original trials that have not been identified by the included reviews increases the potential effect of publication lag and increases the chance that some evidence has not been considered in the review process [10]. However, the inclusion of a recent systematic review [37] that was both comprehensive and of high methodological quality mitigates this issue to a large extent.

Citation analyses performed in this overview indicated that there was overlap between the included reviews. Many interventional studies have contributed to multiple systematic reviews. Therefore, when interpreting the results of this overview, it is important not to treat the included systematic reviews as independent observations, but rather see them as a different way to address similar research questions to determine whether different review teams draw broadly similar conclusions about the effectiveness of HT for patients with chronic HF.

### Implications for Policy and Practice

This overview provides a comprehensive analysis and synthesis that can be used as an evidence map to inform practitioners and policy makers about the effectiveness of HT interventions for patients with HF. Clinicians, health care policy makers, and clinical guideline developers, who rely on systematic reviews and RCTs to help them make informed choices among alternative interventions for the management of HF patients, may use the summary of findings tables of this overview as an entry point to HT evidence. Quality appraisal results can also be used to identify reviews and primary studies of high quality with minimal flaws in both their design and execution that can be trusted to support decision making or address specific questions and details not covered in this overview.

The positive findings associated with HT interventions may present useful resources for policy makers as they address timely issues involving the process and outcomes of care. Mortality, which represents an outcomes measure, has always been used as an indicator of performance and quality of care. Hospital readmissions related to HF also represent a popular indicator of the evolution of a patient’s condition, which is directly linked to the process of care. As such, it can be argued that there is evidence of quality improvement in patient care and a potential alleviation of pressure on hospitals in terms of patient hospitalizations and admissions related to HF conditions, which may otherwise free up places for other patients. Nonetheless, in light of the abovementioned limitations in the quality of evidence of prior research, we concur with Stroetmann et al [66] that making the case for investment in HT applications at a national or international level, requires robust evaluations of the benefits and cost-effectiveness of HT applications under “routine conditions” in different contexts and settings toward the creation of a more convincing evidence base, not only to show that HT works, but also to show in what organizational context it works, for whom, and at what cost. Therefore, from a policy perspective, it is critical to take into consideration the findings of this overview and formulate appropriate policies and funding mechanisms that will support careful evaluations of the socioeconomic impacts of HT in real conditions, greater awareness and exchange of information between key stakeholders about the potential benefits of HT, opportunities for disseminating best-practices, and initiatives that bring policy responsibilities together to support better collaboration and coordination across sectors [66].

Health care decision makers and practitioners who are faced with implementing HT programs in community settings need to consider the complexity of these programs when interpreting the results of the systematic reviews. It is important to recognize that HT technologies are tools that facilitate early detection of deterioration signs. The key to the success of these programs is not the technology itself, but the coordination of care that needs to be in place along the continuum of health services delivered for HF patients within a health care system [62]. The effects of HT will most likely be better when the technology is used as part of a comprehensive and integrated care package that involves various multidisciplinary program components recommended by clinical guidelines [62], for example, patient education, appropriate pharmacological treatment, and psychological support. There is evidence suggesting that tailoring the interventions to those who have been recently discharged from the hospital due to HF exacerbation and are at high risk of sudden death or re-hospitalization may be beneficial to the effectiveness of the treatment strategy. However, health care decision makers should be cautious about implementing these approaches until further evidence accumulates and corroborates these findings.

### Implications for Research

As shown in this overview, there exists a considerable body of evidence evaluating the effectiveness of HT interventions for patients with HF. Researchers conducting both primary studies and systematic reviews should consider the breadth of knowledge that has been created over the years and attempt to address existing gaps in order to inform future deployment and configuration of HT services for patients with HF. For example, new trials should select a small set of potentially mediating variables or risk factors highlighted in previous research studies (eg, HF severity, age, psychological support) and empirically test them within multifactorial designs or, alternatively, explore their impact on outcomes and publish results in meaningful ways as to allow stratification of the benefits of HT programs across subgroups of patients with HF [34]. Future research should also focus on carrying out direct comparisons between different HT technologies and delivery methods to elucidate whether there is differential effectiveness between HT strategies. Collection and reporting of rich contextual information pertaining to the features or components of HT interventions that contribute to variation in outcomes will facilitate a better understanding of the process by which HT works, improve the available evidence base, and maximize the meaningfulness of research findings.

The results from the post-hoc analysis conducted in this overview along with the recent findings of Conway et al [60] have significant implications for future research and provide important methodological insights that need to be considered in conducting future systematic reviews and meta-analyses evaluating the effects of HT interventions. Future systematic reviews should compare the effects of different HT technologies and interventions to provide specific insights on which approaches provide more effective management of HF patients. Development and use of a wide-ranging taxonomy that can adequately classify all types and aspects of HT interventions from the most comprehensive to those that are more simple and selective in what they offer can facilitate more robust comparisons and syntheses of results across studies and can enable interpretation of outcomes with reference to specific monitoring applications and components [64]. It is our hope that the preliminary taxonomy of HT technologies provided in this overview will serve as a valuable starting point toward accomplishing this goal.

Overall, there is a great need to shift our research focus from the basic evaluation question of “is HT effective?” to “what features or components of HT are effective, which patients benefit more from these interventions, under what circumstances, for how long, and why?” This shift requires use of multidisciplinary research designs and methodologies capable of untangling the often complex set of factors that may influence the effects of HT [64]. Realist reviews for instance [67], which attempt to provide an explanatory analysis of how and why complex interventions work (or not) in particular contexts [68,69], can help further advance our conceptual understanding about the impact of human behavior and interactions on the outcomes of telemonitoring interventions.

Finally, given our observation that 80% (12/15) of the systematic reviews assessed in this overview had moderate or major methodological limitations, researchers are strongly encouraged to closely adhere to the available methodological and reporting guidelines for systematic reviews [70-72] and consider the AMSTAR evaluation criteria [16] in order to improve the methodological rigor and reporting quality of their work. Similarly, at the primary study level, more carefully designed trials with longer observation periods, adequate power to detect differences in outcomes, and comprehensive economic evaluations are needed to provide conclusive answers on the effectiveness, cost-effectiveness, viability, and long-term impacts of HT interventions.

### Conclusions

Overviews of systematic reviews use explicit research methods to collect and synthesize in a single source a comprehensive body of published evidence on the effectiveness of interventions. This overview identified and summarized available evidence from 15 systematic reviews on the effectiveness of HT interventions for patients with HF. It also conducted a post-hoc analysis to offer further insights into the various types of HT technologies included in the systematic reviews and investigate the link between HT technologies and HT effectiveness. The results from the principal analysis of this overview suggest that compared with usual care, HT interventions improve survival rates and reduce the risk of HF-related hospitalizations. Patients who have been recently discharged (≤28 days) from an acute care setting and are at high risk of re-hospitalization or sudden death appear to benefit more from HT programs compared to patients with stable HF, but this finding needs to be confirmed in large and rigorously designed RCTs. Overall, the favorable effects of HT reported in previous systematic reviews are based on moderate or low-quality evidence. The results of the post-hoc analyses suggest that only interventions involving automated device-based telemonitoring and mobile telemonitoring are effective in reducing the risk of all-cause mortality and HF-related hospitalizations. However, these findings should be interpreted with caution and be considered as hypothesis generating rather than hypothesis testing due to the exploratory nature of our investigation. More research data are required for interactive voice response systems, video-consultation, and Web-based telemonitoring to provide robust conclusions about their effectiveness. Future research should investigate further which HT strategies provide optimal outcomes, under what circumstances, and for which patient subgroup by adopting multidisciplinary methodologies capable of untangling the often complex set of factors that influence the effects of HT interventions.

## Multimedia Appendix 1

Search strategy.

## Multimedia Appendix 2

AMSTAR operationalization.

## Multimedia Appendix 3

Citation analysis and references of all primary studies included in the systematic reviews.

## Multimedia Appendix 4

Excluded articles and reasons for exclusion.

## Multimedia Appendix 5

Risk of bias assessment in original studies by outcome.

## Multimedia Appendix 6

Post-hoc meta-analyses.

## Abbreviations

AMSTAR: Assessment of Multiple Systematic Reviews
ARR: absolute risk reduction
CI: confidence interval
Crl: credible interval
GRADE: Grading of Recommendations Assessment, Development, and Evaluation
HF: heart failure
HR: hazard ratio
HT: home telemonitoring
PICO: population, intervention, comparison, outcomes
PRISMA: preferred reporting items for systematic reviews and meta-analyses
RCT: randomized controlled trial
RR: risk ratio
