# Supplementary material for: Effects of Home Telemonitoring Interventions on Patients With Chronic Heart Failure: An Overview of Systematic Reviews
Source: J Med Internet Res. 2015 Mar 12;17(3):e63. doi: 10.2196/jmir.4174 (PMC4376138; doi:10.2196/jmir.4174)
Supplement: Supplementary file 1 [file jmir_v17i3e63_app1.pdf]

Appendix 1: Search Strategy

MEDLINE (PubMed)

1. telemedicine[MeSH Terms]
2. home care services[MeSH Terms:noexp]
3. monitoring, ambulatory[MeSH Terms:noexp]
4. monitoring, physiologic[MeSH Terms:noexp]
5. tele med*[Text Word]
6. tele-med*[Text Word]
7. telecare*[Text Word]
8. telemonitor*[Text Word]
9. tele-monitor*[Text Word]
10. teleconsult*[Text Word]
11. telecommunicat*[Text Word]
12. telehealth*[Text Word]
13. telemetry[Text Word]
14. tele-consult*[Text Word]
15. tele-health*[Text Word]
16. telehome[Text Word]
17. tele-home[Text Word]
18. telehomecare[Text Word]
19. tele-homecare[Text Word]
20. telematic[Text Word]
21. telenurs*[Text Word]
22. tele-nurs*[Text Word]
23. remote consult*[Text Word]
24. remote monitoring[Text Word]
25. or/1-24
26. systematic[sb]
27. ("1996/01/01"[PDAT] : "3000"[PDAT])
28. Humans[MeSH Terms])
29. 25 and 26 and 27 and 28

EMBASE (OvidSP)

1. exp telemedicine/
2. home care services/
3. monitoring, ambulatory/
4. monitoring, physiologic/
5. tele med$.tw
6. tele-med$.tw
7. telecare$.tw
8. telemonitor$.tw
9. tele-monitor$.tw
10. teleconsult$.tw
11. telecommunicat$.tw

12. telehealth$.tw
13. telemetry.tw
14. tele-consult$.tw
15. tele-health$.tw
16. telehome.tw
17. tele-home.tw
18. telehomecare.tw
19. tele-homecare.tw
20. telematic.tw
21. telenurs$.tw
22. tele-nurs$.tw
23. remote consult$.tw
24. remote monitoring.tw
25. or/1-24
26. MEDLINE.tw.
27. exp systematic review/ or systematic review.tw
28. meta-analysis/
29. intervention$.ti
30. or/26-29
31. limit 31 to ed=19960101-20131213
32. limit 31 to human
33. 25 and 30 and 31 and 32

CINAHL (EBSCOhost)

1. (MH "Telecommunications") or (MH "Interactive Voice Response Systems") or (MH "Telehealth") or (MH "Telemedicine") or (MH "Remote Consultation") or (MH "Telenursing")
2. TX (tele med*)
3. TX (tele-med*)
4. TX (telemed*)
5. TX (telecare*)
6. TX (telemonitor*)
7. TX (tele-monitor*)
8. TX (teleconsult*)
9. TX (telecommunicat*)
10. TX (telehealth*)
11. TX (telemetry)
12. TX (tele-consult*)
13. TX (tele-health*)
14. TX (telehome)
15. TX (tele-home)
16. TX (telehomecare)
17. TX (tele-homecare)
18. TX (telematic)
19. TX (telenurs*)
20. TX (tele-nurs*)
21. TX (remote consultation)

22. TX (remote monitoring)
23. or/1-22
24. TX (MEDLINE)
25. ((MH "Systematic Review")) OR (TX (systematic review))
26. (MH "Meta Analysis")
27. TI (intervention*)
28. or/24-27
29. 23 and 28
30. *Limiters*: Date of Publication (1996/01/01-2013/12/13); Human; peer-reviewed
31. 29 and 30

Cochrane Library: Cochrane Database of Systematic Reviews, Database of Abstracts of Reviews of Effect (DARE), Health Technology Assessment Database (HTA)

1. MeSH descriptor: [Telemedicine] explode all trees
2. MeSH descriptor: [Home Care Services] this term only
3. MeSH descriptor: [Monitoring, Ambulatory] this term only
4. MeSH descriptor: [Monitoring, Physiologic] this term only
5. tele-med*
6. tele med
7. telecare*
8. telemonitor*
9. tele-monitor*
10. teleconsult*
11. telecommunicat*
12. telehealth*
13. telemetry
14. tele-consult*
15. tele-health*
16. telehome
17. tele-home
18. telehomecare
19. tele-homecare
20. telematic
21. telenurs*
22. tele-nurs*
23. remote consult*
24. remote monitoring
25. or/1-24
