# Supplementary material for: Effects of Home Telemonitoring Interventions on Patients With Chronic Heart Failure: An Overview of Systematic Reviews
Source: J Med Internet Res. 2015 Mar 12;17(3):e63. doi: 10.2196/jmir.4174 (PMC4376138; doi:10.2196/jmir.4174)
Supplement: Supplementary file 3 [file jmir_v17i3e63_app3.pdf]

## Appendix 3: Citation Analysis

As a means of evaluating the comparability and comprehensiveness of the included reviews, as well as the extent to which reviews overlapped in terms of included primary studies, we carried out a citation analysis. More specifically, we generated citation matrices that cross-linked individual systematic reviews identified and included in this overview, with previously published home telemonitoring RCTs (Table 1) and observational studies (Table 2) included in the systematic reviews of our sample.

In order to account for the lag time between systematic review search completion and publication of the review in electronic or print format, we incorporated into the citation matrices the range of search dates reported in each review and their publication date. As shown in Tables 1 and 2, the lag time between search completion and publication is depicted in grey. In reviews that did not report the exact month or day of the search completion for a given year, we used as the end of the search the publication date of the last article that was included in the review.

**Table 1:** Citation matrix of previously published randomized controlled trials inlcluded in the systematic reviews

■ Primary study cited and included in the systematic review
░ Study published after the reported end date of the search for relevant articles
X Study published after publication date of the systematic review

| Author - Publication date | Number of RCTs | Reported search range | [1] | [2] | [3] | [4] | [5] | [6] | [7] | [8] | [9] | [10] | [11] | [12] | [13] | [14] | [15] | [16] | [17] | [18] | [19] | [20] | [21] | [22] | [23] | [24] | [25] | [26] | [27] | [28] | [29] | [30] | [31] | [32] | [33] | [34] | [35] | [36] | [37] | [38] |
|---|---|---|---|---|---|---|---|---|---|---|---|---|---|---|---|---|---|---|---|---|---|---|---|---|---|---|---|---|---|---|---|---|---|---|---|---|---|---|---|---|
| **Louis** et al - Oct 2003 | 7 | 1966 - 2002 | ■ | ■ | ■ | ■ | ■ | ■ | ■ | ░ | ░ | ░ | ░ | X | | | | | | | | | | | | | | | | | | | | | | | | | | |
| **Martinez** et al - July 2006 | 10* | 1966 - Apr 2006 | | ■ | ■ | ■ | ■ | ■ | ■ | ■ | | ■ | | ■ | | ■ | | | ░ | X | | | | | | | | | | | | | | | | | | | | |
| **Chaudhry** et al - Feb 2007 | 4 | 1966 - Aug 2006 | | | | ■ | | ■ | | ■ | | | | | | ■ | | ░ | ░ | ░ | X | | | | | | | | | | | | | | | | | | | |
| **Clark** et al - May 2007 | 5 | Jan 2002 - May 2006** | | | | | ■ | ■ | ■ | | | | | | ■ | ■ | | ░ | ░ | ░ | X | | | | | | | | | | | | | | | | | | | |
| **Paré** et al - May 2007 | 5* | 1990 - 2006 | | | | | ■ | ■ | | ■ | | | | | ■ | ■ | | | | | ░ | ░ | ░ | X | | | | | | | | | | | | | | | | |
| **Seto** - Sep 2008 | 5 | all dates - Nov 2007 | ■ | | | ■ | | | | ■ | | | | ■ | | | | ■ | | | | | | | ░ | X | | | | | | | | | | | | | | |
| **Maric** et al - Mar 2009 | 12 | all dates - Aug 2007 | | | | ■ | ■ | ■ | | ■ | ■ | ■ | ■ | | ■ | ■ | | | ■ | ■ | | | ■ | | ░ | X | | | | | | | | | | | | | | |
| **Dang** et al - Oct 2009 | 9 | 1966 - Apr 2009 | | | | ■ | | ■ | ■ | ■ | | | | | | ■ | | | | ■ | | | | | ■ | | ■ | | | | | ■ | | | ░ | X | | | | |
| **Polisena** et al - Mar 2010 | 11 | 1998 - 2008 | | | | ■ | ■ | ■ | ■ | ■ | | ■ | ■ | | ■ | ■ | | | | | | | | | | | | | | ■ | | ■ | ░ | ░ | ░ | X | | | | |
| **Paré** et al - Apr 2010 | 10* | 1966 - 2008 | | | | | ■ | ■ | ■ | ■ | ■ | | | | | ■ | | | | | | | | | ■ | ■ | | | ■ | ■ | | ░ | ░ | ░ | ░ | X | | | | |
| **Inglis** et al - Aug 2010 | 14 | Jan 2002- Nov 2008*** | | | | | ■ | ■ | ■ | | | | | | ■ | ■ | | | | | ■ | ■ | | ■ | | ■ | | ■ | ■ | | ■ | ■ | ■ | ░ | ░ | ░ | ░ | ░ | X | |
| **Clarke** et al - Jan 2011 | 13 | Jan 1969 - Oct 2009 | | | | | | ■ | ■ | ■ | | | | | | ■ | ■ | | | | | | | | ■ | ■ | ■ | ■ | ■ | | | ■ | ■ | ■ | ░ | ░ | ░ | ░ | X | |
| **Giamouzis** et al - June 2012 | 12 | 2001- Nov 2011 | | | | | | ■ | | | | | | | | ■ | | | | | | | | | | ■ | | | ■ | | | ■ | ■ | ■ | ■ | ■ | | ■ | ■ | ■ |
| **Kraai** et al - August 2011 | 4 | up to Nov 2010 | ■ | | | | | | ■ | | | | | | | | | ■ | | | | | | | | | | | | | | | | | | | ■ | | | |
| **Pandor** et al - August 2013 | 20 | Nov 2008 - Jan 2012**** | | | | | ■ | ■ | ■ | | | | | | ■ | ■ | | | | | ■ | ■ | | ■ | | ■ | | ■ | ■ | | ■ | ■ | ■ | ■ | ■ | | ■ | ■ | ■ | ■ |

* Number of unique RCTs after removal of double-counted studies

** Update of the systematic review by Louis et al (2003)

*** Update of the systematic review by Clark et al (2007)

**** Update of the systematic review by Inglis et al (2010)

**Table 2:** Citation matrix of previously published observational studies inlcluded in the systematic reviews

■ Primary study cited and included in the systematic review
▒ Study published after the end date of the search for relevant articles
X Study published after publication date of study in question

| Author - Publication date | Number of studies | Reported search range | [1] | [2] | [3] | [4] | [5] | [6] | [7] | [8] | [9] | [10] | [11] | [12] | [13] | [14] | [15] | [16] | [17] | [18] | [19] | [20] | [21] | [22] | [23] | [24] | [25] | [26] | [27] | [28] | [29] | [30] | [31] | [32] | [33] | [34] | [35] |
|---|---|---|---|---|---|---|---|---|---|---|---|---|---|---|---|---|---|---|---|---|---|---|---|---|---|---|---|---|---|---|---|---|---|---|---|---|---|
| **Louis** - Oct 2003 | 17 | 1966 - 2002 | | | ■ | ■ | ■ | | | | | ■ | ■ | ■ | ■ | | | | ■ | ■ | | | | | | | | | ■ | ■ | ■ | ■ | ■ | ■ | ■ | ■ | X |
| **Martinez** - July 2006 | 29 | 1966 - Apr 2006 | | ■ | | ■ | ■ | ■ | | | ■ | ■ | ■ | ■ | ■ | | | ■ | ■ | | ■ | ■ | | ■ | | | | | ■ | ■ | | ■ | ■ | | ■ | | ■ |
| **Paré** - May 2007 | 9 | 1990 - 2006 | ■ | | | ■ | | | | | | | | ■ | ■ | | | | | | | ■ | | | | | | | | | | | | | | | |
| **Seto** - Sep 2008 | 5 | all dates - Nov 2007 | | | | | | | | | | | | | | | | | | | | | | | | | | | | | | | | | | | ■ |
| **Maric** - Mar 2009 | 30 | all dates - Aug 2007 | | | | | | | ■ | ■ | ■ | | | ■ | ■ | ■ | ■ | ■ | ■ | ■ | | ■ | ■ | | ■ | ■ | ■ | ■ | | | | | | | | | ■ |
| **Polisena** - Mar 2010 | 10 | 1998 - 2008 | | | | | | | | | | | | ■ | | | | ■ | | | ■ | | | | | ■ | | | | | | | | | | | |
| **Paré** - Apr 2010 | 5 | 1966 - 2008 | | | | | | | | | | | | | | | | ■ | | | | | | | | | | | | | | | | | | | |
| **Kraai** - August 2011 | 10 | up to Nov 2010 | | | | | | | | | ■ | | | | | | | | | | | ■ | | | | | | | | | | | | | | | |

| Author - Publication date | Number of studies | Reported search range | [36] | [37] | [38] | [39] | [40] | [41] | [42] | [43] | [44] | [45] | [46] | [47] | [48] | [49] | [50] | [51] | [52] | [53] | [54] | [55] | [56] | [57] | [58] | [59] | [60] | [61] | [62] | [63] | [64] | [65] | [66] | [67] |
|---|---|---|---|---|---|---|---|---|---|---|---|---|---|---|---|---|---|---|---|---|---|---|---|---|---|---|---|---|---|---|---|---|---|---|
| **Louis** - Oct 2003 | 17 | 1966 - 2002 | | | | | | | | | | | | | | | | | | | | | | | | | | | | | | | | |
| **Martinez** - July 2006 | 29 | 1966 - Apr 2006 | ■ | | | ■ | ■ | ■ | ■ | ■ | ■ | | ■ | | | | ■ | | | | | | | | | | ▒ | ▒ | ▒ | X | | | | |
| **Paré** - May 2007 | 9 | 1990 - 2006 | | | | | | | | | | | ■ | | | | | | ■ | ■ | ■ | | | | | | | | | ▒ | ▒ | X | | |
| **Seto** - Sep 2008 | 5 | all dates - Nov 2007 | | | | | | | | | | | | ■ | | | | | | | ■ | | | | | ■ | | | ■ | | | ▒ | X | |
| **Maric** - Mar 2009 | 30 | all dates - Aug 2007 | ■ | ■ | ■ | | | | | | | | ■ | | ■ | ■ | | ■ | ■ | | | ■ | ■ | | ■ | | ■ | | | ■ | | ▒ | X | |
| **Polisena** - Mar 2010 | 10 | 1998 - 2008 | | | | | | | | | | ■ | ■ | | | | | | ■ | | | | | | | | | ■ | ■ | | | ■ | ▒ | X |
| **Paré** - Apr 2010 | 5 | 1966 - 2008 | | | | | | | | | | | ■ | | | | | | ■ | | ■ | | | | | | ■ | | | | | | ▒ | X |
| **Kraai** - August 2011 | 10 | up to Nov 2010 | ■ | | | ■ | | | | | | | | | | | | | | | | | | ■ | | | ■ | | | | ■ | ■ | ■ | ■ |

**References (Randomized Controlled Trials)**


1. Johnston B, Wheeler L, Deuser J, Sousa KH. Outcomes of the Kaiser Permanente Tele-Home Health Research Project. Archives of family medicine. 2000;**9**:40-5.

2. Massie B, West J, Van Ostaeyen D, *et al.* A controlled trial of heart failure management programs. *J Am Coll Cardiol* 2001;**37**:1A–648A.

3. Bondmass M, Benetar J, Castro G, *et al.* A prospective randomised study comparing outcomes and outpatient care delivery methods for chronic heart failure. *J Am Coll Cardiol* 2001;**37**:1A–648A.

4. Jerant AF, Azari R, Martinez C, Nesbitt TS. A randomized trial of telenursing to reduce hospitalization for heart failure: patient-centered outcomes and nursing indicators. Home health care services quarterly. 2003;**22**:1-20.
   Jerant AF, Azari R, Nesbitt TS. Reducing the cost of frequent hospital admissions for congestive heart failure: a randomized trial of a home telecare intervention. Medical care. 2001;**39**:1234-1245.

5. De Lusignan S, Wells S, Johnson P, Meredith K, Leatham E. Compliance and effectiveness of 1 year's home telemonitoring. The report of a pilot study of patients with chronic heart failure. Eur J Heart Fail. 2001;3(6):723–30.
   de Lusignan S, Althans A, Wells S, Johnson P, Vandenburg M, Robinson J. A pilot study of radiotelemetry for continuous cardiopulmonary monitoring of patients at home. Journal of telemedicine and telecare. 2000;**6 Suppl 1**:S119-22.
   de Lusignan S, Meredith K, Wells S, Leatham E, Johnson P. A controlled pilot study in the use of telemedicine in the community on the management of heart failure--a report of the first three months. Studies in health technology and informatics. 1999;**64**:126-37.

6. Goldberg L, Piette J, Walsh M, Frank T, Jaski B. A daily electronic home monitoring system in patients with advanced heart failure improves survival: The WHARF (Weight Monitoring in Heart Failure) trial. Journal of cardiac failure. 2002;**8**:S54.
   Goldberg LR, Piette JD, Walsh MN, et al. Randomized trial of a daily electronic home monitoring system in patients with advanced heart failure: the Weight Monitoring in Heart Failure (WHARF) trial. American heart journal. 2003;**146**:705-12.

7. Woodend A, Fraser M, Sherrard H, Stueve L. Readmission and quality of life: the impact of telehome care in heart failure. Journal of cardiac failure. 2002;8(4):S98.
   Woodend A, Sherrard H, Fraser M, et al. Getting Connected: Telehome Care for Patients. Canadian Journal of Home Economics. 2003;**52**:19-28.
   Woodend AK, Sherrard H, Fraser M, Stuewe L, Cheung T, Struthers C. Telehome monitoring in patients with cardiac disease who are at high risk of readmission. Heart & lung : the journal of critical care. 2008;**37**:36-45.

8. Benatar D, Bondmass M, Ghitelman J, Avitall B. Outcomes of chronic heart failure. Archives of internal medicine. 2003;**163**:347-52.

**9.** Barnason S, Zimmerman L, Nieveen J, Schmaderer M, Carranza B, Reilly S. Impact of a home communication intervention for coronary artery bypass graft patients with ischemic heart failure on self-efficacy, coronary disease risk factor modification, and functioning. Heart & lung : the journal of critical care. 2003;**32**:147-58.

**10.** Artinian NT, Harden JK, Kronenberg MW, et al. Pilot study of a Web-based compliance monitoring device for patients with congestive heart failure. Heart & lung : the journal of critical care. 2003;**32**:226-33.

**11.** LaFramboise LM, Todero CM, Zimmerman L, Agrawal S. Comparison of Health Buddy with traditional approaches to heart failure management. Family & community health. 2003;**26**:275-88.

**12.** Noel HC, Vogel DC, Erdos JJ, Cornwall D, Levin F. Home telehealth reduces healthcare costs. Telemedicine journal and e-health : the official journal of the American Telemedicine Association. 2004;**10**:170-83.

**13.** Capomolla S, Pinna G, Larovere M, et al. Heart failure case disease management program: a pilot study of home telemonitoring versus usual care. European Heart Journal Supplements. 2004;**6**:F91-F98.

**14.** Cleland JGF, Louis AA, Rigby AS, Janssens U, Balk AHMM. Noninvasive home telemonitoring for patients with heart failure at high risk of recurrent admission and death: the Trans-European Network-Home-Care Management System (TEN-HMS) study. Journal of the American College of Cardiology. 2005;**45**:1654-1664.
Robinson S, Stroetmann K, Stroetmann V. Tele-homecare for chronically ill patients: Improved outcomes and new developments. The Journal on Information Technology in Healthcare. 2004;**2**:251-262.

**15.** Weintraub AR, Kimmelstiel C, Levine D, et al. A Multicenter Randomized Controlled Comparison of Telephonic Disease Management vs. Automated Home Monitoring in Patients Recently Hospitalized with Heart Failure: Span-CHF II Trial. Journal of Cardiac Failure. 2005;**11**:720.
Weintraub A, Gregory D, Patel AR, et al. A multicenter randomized controlled evaluation of automated home monitoring and telephonic disease management in patients recently hospitalized for congestive heart failure: the SPAN-CHF II trial. Journal of cardiac failure. 2010;**16**:285–92.

**16.** Finkelstein SM, Speedie SM, Demiris G, *et al.* Telehomecare: quality, perception, satisfaction. *Telemed J E Health* 2004;**10**:122-8
Finkelstein SM, Speedie SM, Potthoff S. Home telehealth improves clinical outcomes at lower cost for home healthcare. Telemedicine journal and e-health : the official journal of the American Telemedicine Association. 2006;**12**:128-36.

**17.** Spaeder J, Najjar SS, Gerstenblith G, *et al.* Rapid titration of carvedilol in patients with congestive heart failure: a randomized trial of automated telemedicine versus frequent outpatient clinic visits. *Am Heart J* 2006;**151**:e1–10.

Palmer JG, Spaeder JA. Outpatient management of chronic diseases using the telewatch patient monitoring system. *Johns Hopkins APL Tech Dig* 2004;**25**:253–60.

**18.** Kashem A, Droogan MT, Santamore WP, et al. Web-based Internet telemedicine management of patients with heart failure. Telemedicine journal and e-health : the official journal of the American Telemedicine Association. 2006;**12**:439-47.

**19.** Villani A, Malfatto G, Della Rosa F, et al. [Disease management for heart failure patients: role of wireless technologies for telemedicine. The ICAROS project]. Giornale italiano di cardiologia (2006). 2007;**8**:107-14.

**20.** Kielblock B, Frye C, Kottmair S, Hudler T, Siegmund-Schultze E, Middeke M. [Impact of telemetric management on overall treatment costs and mortality rate among patients with chronic heart failure]. Deutsche medizinische Wochenschrift (1946). 2007;**132**:417-22.

**21.** Pinna G, Maestri R, Andrews D, Witkowski T, Capomolla S, Scanferlato JL, Gobbi E, Ferrari M, Ponikowski P, Sleight P, Mortara A, Johnson P. Home telemonitoring of vital signs and cardiorespiratory signals in heart failure patients: system architecture and feasibility of the HHH model. Int J Cardiol 2007;**120**:371-379.
Cleland JGF, Coletta AP, Clark AL. Clinical trials update from the joint European society and world congress of cardiology meeting: PEP-CHF, ACCLAIM and the HHH study. Eur J Heart Fail 2006;8:658–661.
Doehner W. World Congress of Cardiology 2006: Barcelona, Spain, 2–5 September 2006. Heart Fail Monit 2007;5:97–100.
Mortara A, Pinna GD, Johnson P, La Rovere MT, Ponikowski P, Tavazzi L, Sleight P. A multi-country randomised trial of the role of a new telemonitoring system in CHF: the HHH study (Home or Hospital in Heart Failure). Rational, study design and protocol. Eur Heart J Suppl 2004;6(Suppl. F):F99–F102.

**22.** Blum K, Gottlieb SS. Morbidity and Mortality Benefits of Reliable Instrumental Support. Journal of Cardiac Failure. 2007;**13**:S164.

**23.** Dansky KH, Vasey J, Bowles K. Impact of telehealth on clinical outcomes in patients with heart failure. Clinical nursing research. 2008;**17**:182-99.

**24.** Antonicelli R, Mazzanti I, Abbatecola AM, Parati G. Impact of home patient telemonitoring on use of β-blockers in congestive heart failure. Drugs & aging. 2010;**27**:801-05.
Antonicelli R, Testarmata P, Spazzafumo L, et al. Impact of telemonitoring at home on the management of elderly patients with congestive heart failure. Journal of telemedicine and telecare. 2008;**14**:300-305.

**25.** Wakefield BJ, Ward MM, Holman JE, et al. Evaluation of home telehealth following hospitalization for heart failure: a randomized trial. Telemedicine journal and e-health : the official journal of the American Telemedicine Association. 2008;**14**:753-61.

**26.** Balk AH, Davidse W, Dommelen Pv, et al. Tele-guidance of chronic heart failure patients enhances knowledge about the disease. A multi-centre, randomised controlled study. European journal of heart failure. 2008;**10**:1136-42.

**27.** Soran OZ, Piña IL, Lamas GA, et al. A randomized clinical trial of the clinical effects of enhanced heart failure monitoring using a computer-based telephonic monitoring system in older minorities and women. Journal of cardiac failure. 2008;**14**:711-17.

**28.** Schwarz KA, Mion LC, Hudock D, Litman G. Telemonitoring of heart failure patients and their caregivers: a pilot randomized controlled trial. Progress in cardiovascular nursing. 2008;**23**:18-26.

**29.** Zugck C, Frankeinstein L, Nelles M, et al. Telemedicine reduces hospitalisation rates in patients with chronic heart failure - results of the randomized HiTel trial. European Journal of Heart Failure Supplements. 2008;**7**:9.

**30.** Giordano A, Scalvini S, Zanelli E, et al. Multicenter randomised trial on home-based telemanagement to prevent hospital readmission of patients with chronic heart failure. International journal of cardiology. 2009;**131**:192-99.
Scalvini S, Zanelli E, Volterrani M, Benigno M. Effect of a home based telecardiology on chronic heart failure outcomes: a case control pilot study. ESC Congress. 2002, 31 August – 4 September, Berlin, Germany.

**31.** Mortara A, Pinna GD, Johnson P, et al. Home telemonitoring in heart failure patients: the HHH study (Home or Hospital in Heart Failure). European journal of heart failure. 2009;**11**:312-18.

**32.** Dar O, Riley J, Chapman C, et al. A randomized trial of home telemonitoring in a typical elderly heart failure population in North West London: results of the Home-HF study. European journal of heart failure. 2009;**11**:319-25.

**33.** Scherr D, Kastner P, Kollmann A, et al. Effect of home-based telemonitoring using mobile phone technology on the outcome of heart failure patients after an episode of acute decompensation: randomized controlled trial. Journal of medical Internet research. 2009;**11**:e34.

**34.** Tompkins C, Orwat J. A randomized trial of telemonitoring heart failure patients. Journal of healthcare management / American College of Healthcare Executives. 2010;**55**:312-22.

**35.** Kulshreshtha A, Kvedar JC, Goyal A, *et al.* Use of remote monitoring to improve outcomes in patients with heart failure: a pilot trial. *Int J Telemed Appl* 2010;**2010**:870959.

**36.** Wade MJ, Desai AS, Spettell CM, et al. Telemonitoring with case management for seniors with heart failure. The American journal of managed care. 2011;**17**:e71-9.

**37.** Koehler F, Winkler S, Schieber M, et al. Impact of remote telemedical management on mortality and hospitalizations in ambulatory patients with chronic heart failure: the telemedical interventional monitoring in heart failure study. Circulation*.* 2011;**123**:1873-80.

**38.** Dendale P, De Keulenaer G, Troisfontaines P, et al. Effect of a telemonitoring-facilitated collaboration between general practitioner and heart failure clinic on mortality and rehospitalization rates in severe heart failure: the TEMA-HF 1 (TElemonitoring in the MAnagement of Heart Failure) study. European journal of heart failure*.* 2012;**14**:333-40.

**References (Observational Studies)**

**1.** Vincent JA, Cavitt DL, Karpawich PP. Diagnostic and Cost Effectiveness of Telemonitoring the Pediatric Pacemaker Patient. Pediatric Cardiology*.* 1997;**18**:86-90.

**2.** Roth A, Carthy Z, Benedek M. Telemedicine in emergency home care--the "Shahal" experience. *J Telemed Telecare* 1997;**3 Suppl 1**:58–60.

**3.** Roglieri JL, Futterman R, McDonough KL, et al. Disease management interventions to improve outcomes in congestive heart failure. The American journal of managed care*.* 1997;**3**:1831-39.

**4.** Shah NB, Der E, Ruggerio C, Heidenreich PA, Massie BM. Prevention of hospitalizations for heart failure with an interactive home monitoring program. American heart journal*.* 1998;**135**:373-78.

**5.** Williams RE, Keiler L, Sprang M. Telemanagement of congestive heart failure: results of daily weight and symptom tracking. American College of Cardiology Annual Scientific Session. 1998. Abstract: 977-177.
Williams R, Willyard D, Wickemeyer W, *et al.* Clinical outcomes and satisfaction improve over time using chronic heart failure tel-assurance remote patient monitoring: second year results across a statewide system. *J Card Fail* 2004;**10**:111.

**6.** Ruggerio C, Heidenreich P, Massie B, *et al.* Interactive home monitoring and education program for heart failure shows improvement in quality of life. *J Card Fail* 1998;**4**:57.

**7.** Anonymous. Timely access to patient information cuts CHF costs. *Healthc Demand Dis Manag* 1999;**5**:14–5.

**8.** Cross M. A scale that talks back. *Health Data Manag* 1999;**7**:76–8.

**9.** Baer CA, Di Salvo TG, Cail MI, *et al.* Electronic home monitoring of congestive heart failure patients: design and feasibility. *Congest Heart Fail* 1999;**5**:105–13.

**10.** Bondmass M, Malhotra V, Castro G, Avitall B. The long term effect of a telemedicine intervention on heart failure admissions and length of stay. Journal of Cardiac Failure. 1999;**5**:78.

**11.** Chrysogelos ET, Gemme D, Coleman K, Chung ES, Meyer TE. Telemonitoring devices further improve outcomes of a multidisciplinary heart failure outpatient program. Journal of Cardiac Failure. 1999;**5**:73.

**12.** Cordisco ME, Benjaminovitz A, Hammond K, Mancini D. Use of telemonitoring to decrease the rate of hospitalization in patients with severe congestive heart failure. The American journal of cardiology. 1999;**84**:860-62.

**13.** Heidenreich PA, Ruggerio CM, Massie BM. Effect of a home monitoring system on hospitalization and resource use for patients with heart failure. American heart journal. 1999;**138**:633-40.

**14.** OReilly M. Is Internet-based disease management on the way? *Can Med Assoc J* 1999;**160**:1039–1039.

**15.** Knox D, Mischke L. Implementing a congestive heart failure disease management program to decrease length of stay and cost. *J Cardiovasc Nurs* 1999;**14**:55–74

**16.** Bondmass M, Bolger N, Castro G, *et al.* The effect of physiologic home monitoring and telemanagement on chronic heart failure outcomes. *Internet J Adv Nurs Pract* 1999;**3(2)**.

**17.** de Lusignan S, Althans A, Wells S, *et al.* A pilot study of radiotelemetry for continuous cardiopulmonary monitoring of patients at home. *J Telemed Telecare* 2000;**6 Suppl 1**:S119–22.

**18.** Lapworth DJ, Dibiase A. Decreased hospitalizations using a home based electronic weight monitoring system as part of a comprehensive heart failure program. *J Card Fail* 2000;**6**:69.

**19.** Mehra MR, Uber PA, Chomsky DB, *et al.* Emergence of electronic home monitoring in chronic heart failure: rationale, feasibility, and early results with the HomMed Sentry-Observer system. *Congest Heart Fail* 2000;**6**:137–9.

**20.** Nanevicz T, Piette J, Zipkin D, *et al.* The feasibility of a telecommunications service in support of outpatient congestive heart failure care in a diverse patient population. *Congest Heart Fail* 2000;**6**:140–5.
Nanevicz T, Piette J, Zipkin D, *et al.* Feasibility of a home telemonitoring system for prevention of congestive heart failure exacerbation. *J Card Fail* 1999;**5**:63.

**21.** Cherry JC, Colliflower SJ, Tsiperfal A. Meeting the challenges of case management with remote patient monitoring technology. *Lippincotts Case Manag* 2000;**5**:191–8.

**22.** Jenkins RL, McSweeney M. Assessing elderly patients with congestive heart failure via in-home interactive telecommunication. *J Gerontol Nurs* 2001;**27**:21–7.

**23.** Downey C. Disease management uses web to net savings. *Manag Care Mag [online]* 2001;July.

**24.** Vaccaro J, Cherry J, Harper A, *et al.* Utilization Reduction, Cost Savings, and Return on Investment for the PacifiCare Chronic Heart Failure Program, "Taking Charge of Your Heart Health."*Dis Manag* 2001;**4**:131–42.

**25.** Anonymous. Hospital partners with visiting nurses to support CHF telemonitoring. *Qual Lett Healthc Lead* 2002;**14**:10.

**26.** Mueller TM, Vuckovic KM, Knox DA, *et al.* Telemanagement of heart failure: a diuretic treatment algorithm for advanced practice nurses. *Heart Lung* 2002;**31**:340–7.

**27.** Deering M, Baines B, Christianson C, *et al.* Patients and providers evaluate daily home weight and symptom monitoring for CHF management. *J Card Fail* 2002;**8**:S97.

**28.** Ertle D, Litman G. Hospital outpatient disease management initiative achieves marked reduction in inpatient admissions and costs for congestive heart failure. *J Card Fail* 2002;**8**:S90.

**29.** Feldman C, Milstein S, Cinnamond M, *et al.* A protocol driven diuretic dosing system based on bioelectric impedance reduces acute episodes of fluid overload in patients with severe heart failure while minimizing physician management time. *J Card Fail* 2002;**8**:S84.

**30.** Knox D., Mueller T., Vuckovic KM, *et al.* Remote titration of beta-blocker therapy for heart failure by advanced practice nurses, titration protocols, and daily patient telemanagment. *J Card Fail* 2002;**8**:S83.

**31.** Kesinger T, Gilani S, Jennison S. Electronic home monitoring reduces hospital admission, length of stay and readmission frequency in a selected heart failure population. *J Card Fail* 2002;**8**:S94.

**32.** Scalvini S, Zanelli E, Volterrani M, *et al.* Effect of a home based telecardiology on chronic heart failure outcomes: a case control pilot study. *Eur J Heart Fail* 2002;**1:72**.

**33.** Macropoulos LR, Knoop JD. CHF hospital admissions reduced by 57% (0.234 PPPY) in medicare population and 48% (0.299 PPPY) in commercial population using advanced home monitoring program in large patient population. *J Card Fail* 2002;**9**:S103.

**34.** Wang L, Yu C-M, Chau E, *et al.* Feasibility of predicting CHF hospitalization using pacemaker-based impedance sensor in CHF patients. *J Card Fail* 2002;**8**:S81.

**35.** Dimmick SL, Burgiss SG, Robbins S, *et al.* Outcomes of an integrated telehealth network demonstration project. *Telemed J E Health* 2003;**9**:13–23.

**36.** Nobel JJ, Norman GK. Emerging information management technologies and the future of disease management. Dis Manag 2003; **6**(4):219–31.

**37.** Delgado DH, Costigan J, Wu R, *et al.* An interactive Internet site for the management of patients with congestive heart failure. *Can J Cardiol* 2003;**19**:1381–5.

**38.** Chetney R. The Cardiac Connection program: home care that doesn't miss a beat. *Home Healthc Nurse* 2003;**21**:680–6.

**39.** Kobb R, Hoffman N, Lodge R, *et al.* Enhancing elder chronic care through technology and care coordination: report from a pilot. *Telemed J E Health* 2003;**9**:189–95.

**40.** Celler BG, Lovell NH, Basilakis J. Using information technology to improve the management of chronic disease. *Med J Aust* 2003;**179**:242–6.

**41.** Jimison H, Pavel M. Monitoring of body weight for heart failure patients: variability of weight and self-reporting. In: *Proceedings of the 25th Annual International Conference of the IEEE EMBS*. 2003. 3665–8.

**42.** Pinna GD, Maestri R, Roma M, *et al.* Home telemonitoring of chronic heart failure patients: novel system architecture of the home or hospital in heart failure study. In: *Computers in Cardiology, 2003*. IEEE 2003. 105–8.

**43.** Cheitlin J, Guevara R. Providing quality care using remote patient monitoring technology: Mercy Health Center case study. Presentations *9th Annual Meeting and Exposition of the American Telemedicine Association*. 2004.

**44.** Chumbler N, Ryan P, Neugaard B, *et al.* Providing heart smart care through technology and care coordinatione. *Presentations ninth Annual Meeting and Exposition of the American Telemedicine Association*. 2004.

**45.** McManus SG. A Telehealth Program to Reduce Readmission Rates Among Heart Failure Patients: One Agency's Experience. *Home Health Care Manag Pract* 2004;**16**:250–4.

**46.** Roth A, Kajiloti I, Elkayam I, *et al.* Telecardiology for patients with chronic heart failure: the "SHL" experience in Israel. *Int J Cardiol* 2004;**97**:49–55.

**47.** Bradford WD, Kleit AN, Krousel-Wood MA, *et al.* Willingness to pay for telemedicine assessed by the double-bounded dichotomous choice method. *J Telemed Telecare* 2004;**10**:325–30.
Bradford WD, Kleit A, Krousel-Wood MA, *et al.* Comparing willingness to pay for telemedicine across a chronic heart failure and hypertension population. *Telemed J E Health* 2005;**11**:430–8.

**48.** Schneider NM. Managing congestive heart failure using home telehealth. *Home Healthc Nurse* 2004;**22**:719–22.

**49.** Santamore WP, Homko C, Marble J, *et al.* Improving heart failure care by using a telemedicine system. *Conf Proc IEEE Eng Med Biol Soc* 2004;**4**:3076–9.

**50.** Scalvini S, Zanelli E, Volterrani M, *et al.* A pilot study of nurse-led, home-based telecardiology for patients with chronic heart failure. *J Telemed Telecare* 2004;**10**:113–7.

**51.** Wu RC, Delgado D, Costigan J, *et al.* Pilot study of an Internet patient-physician communication tool for heart failure disease management. *J Med Internet Res* 2005;**7**:e8.

**52.** Schofield RS, Kline SE, Schmalfuss CM, *et al.* Early outcomes of a care coordination-enhanced telehome care program for elderly veterans with chronic heart failure. *Telemed J E Health* 2005;**11**:20–7.

**53.** Kjellström B, Igel D, Abraham J, *et al.* Trans-telephonic monitoring of continuous haemodynamic measurements in heart failure patients. *J Telemed Telecare* 2005;**11**:240–4.

**54.** Scalvini S, Capomolla S, Zanelli E, *et al.* Effect of home-based telecardiology on chronic heart failure: costs and outcomes. *J Telemed Telecare* 2005;**11 Suppl 1**:16–8.

**55.** Hudson LR, Hamar GB, Orr P, *et al.* Remote physiological monitoring: clinical, financial, and behavioral outcomes in a heart failure population. *Dis Manag* 2005;**8**:372–81.

**56.** Maglaveras N, Chouvarda I, Koutkias VG, *et al.* The Citizen Health System (CHS): a modular medical contact center providing quality telemedicine services. *IEEE Trans Inf Technol Biomed* 2005;**9**:353–62.

**57.** Walsh M, Coleman JR. Developing a pilot telehealth program: one agency's experience. *Home Healthc Nurse* 2005;**23**:188–91.

**58.** Dang S, Ma F, Nedd N, *et al.* Differential resource utilization benefits with Internet-based care coordination in elderly veterans with chronic diseases associated with high resource utilization. *Telemed J E Health* 2006;**12**:14–23.

**59.** Lehmann CA, Mintz N, Giacini JM. Impact of Telehealth on Healthcare Utilization by Congestive Heart Failure Patients. *Dis Manag Heal Outcomes* 2006;**14**.

**60.** Scherr D, Zweiker R, Kollmann A, *et al.* Mobile phone-based surveillance of cardiac patients at home. *J Telemed Telecare* 2006;**12**:255–61.

**61.** Quinn C. Low-technology heart failure care in home health: improving patient outcomes. *Home Healthc Nurse* 2006;**24**:533–40.

**62.** Myers S. Impact of Home-Based Monitoring on the Care of Patients with Congestive Heart Failure. *Home Health Care Manag Pract* 2006;**18**:444–51.

**63.** Anonymous. Telehealth helps hospital cut readmissions by 75%. *Healthcare Benchmarks Qual Improv* 2007;**14**:92–4.

**64.** Whitten P, Mickus M. Home telecare for COPD/CHF patients: outcomes and perceptions. *J Telemed Telecare* 2007;**13**:69–73.

**65.** Seibert PS, Whitmore TA, Patterson C, *et al.* Telemedicine facilitates CHF home health care for those with systolic dysfunction. *Int J Telemed Appl* 2008;235031.

**66.** Whitten P, Bergman A, Meese MA, *et al.* St. Vincent's Home telehealth for congestive heart failure patients. *Telemed J E Health* 2009;**15**:148–53.

**67.** Cardozo L, Steinberg J. Telemedicine for recently discharged older patients. *Telemed J E Health* 2010;**16**:49–55.
