# Supplementary material for: Effects of Home Telemonitoring Interventions on Patients With Chronic Heart Failure: An Overview of Systematic Reviews
Source: J Med Internet Res. 2015 Mar 12;17(3):e63. doi: 10.2196/jmir.4174 (PMC4376138; doi:10.2196/jmir.4174)
Supplement: Supplementary file 4 [file jmir_v17i3e63_app4.pdf]

Appendix 4: Excluded articles

Author	Reasons for exclusion
Adams 2001	Contra to protocol: not peer-reviewed
Atteberry 2009	Contra to protocol (P): included studies that involved participants with other conditions. Findings were not reported separately for heart failure
Augustin 2012	Contra to protocol (L): non-English review, published in German
Barlow 2007	Contra to protocol (P): Contra to protocol: included studies that involved participants with other conditions (e.g. frail elderly people). Interventions included telephone and web-based systems; findings about home telemonitoring for patients with heart failure stated were not reported separately and results could not be teased out
Barnason 2012	Contra to protocol (I): included studies with other than home telemonitoring interventions (e.g. telephone support and in person one-to-one or group counselling and education sessions)
Bensink 2006	Contra to protocol (P, I): included studies that involved participants with other conditions. Findings were not reported separately for heart failure. Included studies with other than home telemonitoring interventions
Bensink 2007	Contra to protocol (P, I): included studies that involved participants with other conditions. Findings were not reported separately for heart failure. Included studies with other than home telemonitoring interventions
Bowles 2007	Contra to protocol (S): not a systematic review
Currell 2000	Contra to protocol (P, I): included studies that involved participants with other conditions; included studies with other than home telemonitoring interventions
Currell 2001	Contra to protocol (P, I): included studies that involved participants with other conditions; included studies with other than home telemonitoring interventions
Delgado-Passler 2006	Contra to protocol (S): Not a systematic review
DelliFraine 2008	Contra to protocol (P, I): included studies that involved participants with other conditions (e.g. arthritis). Included studies with other than home telemonitoring interventions (e.g. telephone support). Findings were not reported separately
Durrani 2009	Contra to protocol (P, I): included studies that involved participants with other conditions; included studies with other than home telemonitoring interventions (e.g. telephone support). Findings were not reported separately
Eland-de Kok 2011	Contra to protocol (P, I): included studies that involved participants with other conditions (e.g. atopic dermatitis); included studies with other than home telemonitoring interventions (e.g. web-based rehabilitation programs)
Flynn 2010	Contra to protocol (S): Not a systematic review of primary interventional studies
Free 2013a	Contra to protocol (P, I): included studies that involved participants with other conditions; included studies with mobile self-management interventions

Free 2013b	Contra to protocol (P, I): included studies that did not target patients with chronic conditions; included studies with interventions for health services support (e.g. appointment reminders, clinical diagnosis and management).
Gaikwad 2009	Contra to protocol (P, I): included studies that involved participants with other conditions (e.g. patients awaiting surgery for anterior cruciate ligament deficiency); included studies with other than home telemonitoring interventions (e.g. home-based physiotherapy interventions and multidisciplinary interventions)
Garcia Lizana 2009	Contra to protocol (I): included studies with other than home telemonitoring interventions (e.g. interactive multimedia computer games, computer-assisted instructional games)
Garcia Lizana 2007	Contra to protocol (P, I): included studies that involved participants with other conditions. Included studies with other than home telemonitoring interventions (e.g. interactive multimedia computer games, computer-assisted instructional games)
Gonseth 2004	Contra to protocol (I): Not a systematic review of home telemonitoring interventions
Gurman 2012	Contra to protocol (P): included studies that involved participants with other conditions (e.g. HIV, tuberculosis);
Gustafsson 2004	Contra to protocol (I): Not a systematic review of home telemonitoring interventions
Hailey 1997	Contra to protocol (P, I): focused on telemedicine and areas outside the scope of our study
Hailey 2001	Contra to protocol (P, I): focused on telemedicine and areas outside the scope of our study (e.g. mental health, ophthalmology)
Hailey 2002	Contra to protocol (P, I): focused on telemedicine and areas outside the scope of our study (e.g. geriatric care, dermatology)
Hailey 2004	Contra to protocol (P, I): focused on telemedicine and areas outside the scope of our study (e.g. geriatric care, dermatology)
Hakansson 2000	Contra to protocol (S): Not a systematic review
Hayes 2008	Contra to protocol (I): focused on telemedicine and areas outside the scope of our study
Hersh 2002	Contra to protocol (I): Not a systematic review of home telemonitoring interventions
Hersh 2006a	Contra to protocol (P): included studies that involved participants with other conditions (e.g. spinal cord injury)
Hersh 2006b	Contra to protocol (P): included studies that involved participants with other conditions (e.g. spinal cord injury)
Hersh 2001	Contra to protocol (P): focused on patients with other chronic diseases
Jennet 2003	Contra to protocol (P): focused on the application of telehealth in other areas outside the scope of our study (e.g. geriatric care); included studies that involved participants with other conditions (e.g. wound care)
Klersy 2009	Contra to protocol (I): included studies with other interventions (i.e. structured telephone support) besides home telemonitoring; findings from home telemonitoring interventions were not reposted separately, but were combined with findings from structured telephone support

Klersy 2011	Contra to protocol (I): included studies with other interventions (i.e. structured telephone support) besides home telemonitoring; findings from home telemonitoring interventions were not reposted separately (i.e. they were combined with findings from structured telephone support)
Kleinpell 2005	Contra to protocol (S): Not a systematic review
Lindberg 2013	Contra to protocol (P, I): included studies that involved participants with other conditions (coronary artery bypass graft, wound care); included studies with other interventions besides home telemonitoring (e.g., tele-rehabilitation)
McAlister 2004	Contra to protocol (I): included studies with other interventions (e.g. telephone support and multidisciplinary interventions)
Mistry 2012	Contra to protocol (P, I): included studies that focused on telemedicine (clinics/consultations, store and forward technologies) and areas such as dermatology
Mitchell 2011	Could not be retrieved
Neubeck 2009	Contra to protocol (I): included other interventions besides home telemonitoring that did not involve electronic transmission of patient data (e.g., stand-alone telephone support)
Polisena 2009	Contra to protocol (P, I): included studies that involved participants with other conditions and other interventions besides home telemonitoring that do not involve electronic transmission of patient data (e.g., stand-alone telephone support). Findings and conclusions on the effects of home telemonitoring interventions on patients with heart failure were not reported separately and could not be teased out
Radhakrishnan 2012	Contra to protocol (I): included studies with other interventions besides home telemonitoring that do not involve electronic transmission of patient data (e.g., stand-alone telephone support); findings and conclusions from home telemonitoring interventions were not reported separately and could not be teased out
Roccaforte 2005	Contra to protocol (I): Not a systematic review of home telemonitoring interventions
Rojas 2008	Contra to protocol (P, I): included studies that involved participants with other conditions. Included studies with other interventions besides home telemonitoring (e.g., stand-alone telephone support). Findings were not reported separately.
Schmidt 2010	Contra to protocol (S): not a systematic review
Tao 2013	Contra to protocol (P): Patients with diabetes
van Den Berg 2012	Contra to protocol (P, I): included studies that involved participants with other conditions (e.g. cancer, nervous system disorders); included studies with other interventions besides home telemonitoring that do not involve electronic transmission of patient data (e.g., stand-alone telephone). Findings and conclusions about the effects of home telemonitoring interventions on patients with heart failure were not reported separately and could not be teased out
Ward 2012	Contra to protocol (I): Not a systematic review of home telemonitoring interventions

Whellan 2005	Contra to protocol (I): Not a systematic review of home telemonitoring interventions
Whitten 2000	Contra to protocol (I): review focused on teleradiology and other interventions beyond the scope of this overview
Whitten 2002	Contra to protocol (I): review focused on telemedicine interventions in settings and with participants beyond the scope of this overview
Wooton 2012	Contra to protocol (I): included studies with other interventions besides home telemonitoring that do not involve electronic transmission of patient data (e.g., stand-alone telephone support); findings and conclusions about home telemonitoring interventions were not reported separately and could not be teased out for each chronic disease individually
Xiang 2013	Contra to protocol (I): included studies with structured telephone support besides home telemonitoring; findings about home telemonitoring interventions were not analyzed separately

References of excluded articles:

1 Adams E. Physiologic telemonitoring in CHF. *VA Technol Assess Program, Short Rep* 2001;**5**:1–11

2 Atteberry G. The Effects of Telehomecare on Quality and Agency Revenue: A Literature Review. *Home Health Care Manag Pract* 2008;**21**:188–94

3 Augustin U, Henschke C. [Does telemonitoring lead to health and economic benefits in patients with chronic heart failure? - a systematic review]. *Gesundheitswesen* 2012;**74**:e114–21

4 Barlow J, Singh D, Bayer S, *et al.* A systematic review of the benefits of home telecare for frail elderly people and those with long-term conditions. *J Telemed Telecare* 2007;**13**:172–9

5 Barnason S, Zimmerman L, Young L. An integrative review of interventions promoting self-care of patients with heart failure. *J Clin Nurs* 2012;**21**:448–75

6 Bensink M, Hailey D, Wootton R. A systematic review of successes and failures in home telehealth: preliminary results. *J Telemed Telecare* 2006;**12**:8–16

7 Bensink M, Hailey D, Wootton R. A systematic review of successes and failures in home telehealth. Part 2: Final quality rating results. *J Telemed Telecare* 2007;**13**:10–4

8 Bowles KH, Baugh AC. Applying research evidence to optimize telehomecare. *J Cardiovasc Nurs*;**22**:5–15

9 Currell R, Urquhart C, Wainwright P, *et al.* Telemedicine versus face to face patient care: effects on professional practice and health care outcomes. *Cochrane database Syst Rev* 2000:CD002098

10 Currell R, Urquhart C, Wainwright P, *et al.* Telemedicine versus face to face patient care: effects on professional practice and health care outcomes. *Nurs Times* 2001;**97**:35

11 Delgado-Passler P, McCaffrey R. The influences of postdischarge management by nurse practitioners on hospital readmission for heart failure. J Am Acad Nurse Pract 2006;18:154–160

12 Dellifraine JL, Dansky KH. Home-based telehealth: a review and meta-analysis. *J Telemed Telecare* 2008;**14**:62–6

13 Durrani H, Khoja S. A systematic review of the use of telehealth in Asian countries. *J Telemed Telecare* 2009;**15**:175–81

14 Eland-de Kok P, van Os-Medendorp H, Vergouwe-Meijer A, *et al.* A systematic review of the effects of e-health on chronically ill patients. *J Clin Nurs* 2011;**20**:2997–3010

15 Flynn K. Home telehealth. *VA Technol Assess Progr* Published Online First: 2010.http://onlinelibrary.wiley.com/o/cochrane/clhta/articles/HTA-32010001501/frame.html

16 Free C, Phillips G, Galli L, *et al.* The effectiveness of mobile-health technology-based health behaviour change or disease management interventions for health care consumers: a systematic review. *PLoS Med* 2013a;**10**:e1001362

17 Free C, Phillips G, Watson L, *et al.* The effectiveness of mobile-health technologies to improve health care service delivery processes: a systematic review and meta-analysis. *PLoS Med* 2013b;**10**:e1001363

18 Gaikwad R, Warren J. The role of home-based information and communications technology interventions in chronic disease management: a systematic literature review. *Health Informatics J* 2009;**15**:122–46

19 García-Lizana F, Sarría-Santamera A. New technologies for chronic disease management and control: a systematic review. *J Telemed Telecare* 2007;**13**:62–8

20 García-Lizana F, Yanes Lopez V. Information and communications technologies in CHF management programmes. Organizational challenges and evidence status. Agencia de Evaluacion de Tecnologias Sanitarias (AETS) 2009. http://onlinelibrary.wiley.com/o/cochrane/clhta/articles/HTA-32011000305/frame.html

21 Gonseth J, Guallar-Castillón P, Banegas JR, *et al.* The effectiveness of disease management programmes in reducing hospital re-admission in older patients with heart failure: a systematic review and meta-analysis of published reports. *Eur Heart J* 2004;**25**:1570–95

22 Gurman TA, Rubin SE, Roess AA. Effectiveness of mHealth behavior change communication interventions in developing countries: a systematic review of the literature. *J Health Commun* 2012;**17 Suppl 1**:82–104

23 Gustafsson F, Arnold JMO. Heart failure clinics and outpatient management: review of the evidence and call for quality assurance. *Eur Heart J* 2004;**25**:1596–604

24 Hailey D, Jacobs P. Assessment of telehealth applications. Alberta Heritage Foundation for Medical Research (AHFMR) 1997.

25 Hailey D, Roine R, Ohinmaa A. Assessment of telemedicine applications - an update. Alberta Heritage Foundation for Medical Research (AHFMR) 2001.

26 Hailey D, Ohinmaa A, Roine R. Study quality and evidence of benefit in recent assessments of telemedicine. *J Telemed Telecare* 2004;**10**:318–24

27 Hailey D, Roine R, Ohinmaa A. Systematic review of evidence for the benefits of telemedicine. *J Telemed Telecare* 2002;**8 Suppl 1**:1–30

28 Håkansson S, Gavelin C. What do we really know about the cost-effectiveness of telemedicine? *J Telemed Telecare* 2000;**6 Suppl 1**:S133–6

29 Hayes Inc. Remote monitoring of patients with congestive heart failure. HAYES, Inc 2008

30 Hersh WR, Helfand M, Wallace J, *et al.* Clinical outcomes resulting from telemedicine interventions: a systematic review. *BMC Med Inform Decis Mak* 2001;**1**:5

31 Hersh W, Helfand M, Wallace J, *et al.* A systematic review of the efficacy of telemedicine for making diagnostic and management decisions. *J Telemed Telecare* 2002;**8**:197–209. doi:10.1258/135763302320272167

32 Hersh WR, Hickam DH, Severance SM, *et al.* Telemedicine for the medicare population: update. *Evid Rep Technol Assess (Full Rep)* 2006a:1–41

33 Hersh WR, Hickam DH, Severance SM, *et al.* Diagnosis, access and outcomes: Update of a systematic review of telemedicine services. *J Telemed Telecare* 2006b;**12 Suppl 2**:S3–31

34 Jennett PA, Affleck Hall L, Hailey D, *et al.* The socio-economic impact of telehealth: a systematic review. *J Telemed Telecare* 2003;**9**:311–20

35 Kleinpell R, Avitall B. Telemanagement in Chronic Heart Failure. *Dis Manag Heal Outcomes* 2005;**13**:43–52

36 Klersy C, De Silvestri A, Gabutti G, *et al.* Economic impact of remote patient monitoring: an integrated economic model derived from a meta-analysis of randomized controlled trials in heart failure. *Eur J Heart Fail* 2011;**13**:450–9

37 Klersy C, De Silvestri A, Gabutti G, *et al.* A meta-analysis of remote monitoring of heart failure patients. *J Am Coll Cardiol* 2009;**54**:1683–94

38 Lindberg B, Nilsson C, Zotterman D, *et al.* Using Information and Communication Technology in Home Care for Communication between Patients, Family Members, and Healthcare Professionals: A Systematic Review. *Int J Telemed Appl* 2013;**2013**:461829

39 McAlister FA, Stewart S, Ferrua S, *et al.* Multidisciplinary strategies for the management of heart failure patients at high risk for admission: a systematic review of randomized trials. *J Am Coll Cardiol* 2004;**44**:810–9

40 Mistry H. Systematic review of studies of the cost-effectiveness of telemedicine and telecare. Changes in the economic evidence over twenty years. *J Telemed Telecare* 2012;**18**:1–6

41 Mitchell MD, Norris A, Umscheid CA. Telemedicine for patients with congestive heart failure. Center for Evidence-based Practice (CEP) 2011

42 Neubeck L, Redfern J, Fernandez R, *et al.* Telehealth interventions for the secondary prevention of coronary heart disease: a systematic review. *Eur J Cardiovasc Prev Rehabil* 2009;**16**:281–9

43 Polisena J, Coyle D, Coyle K, *et al.* Home telehealth for chronic disease management: a systematic review and an analysis of economic evaluations. *Int J Technol Assess Health Care* 2009;**25**:339–49

44 Radhakrishnan K, Jacelon C. Impact of telehealth on patient self-management of heart failure: a review of literature. *J Cardiovasc Nurs* 2012;**27**:33–43

45 Roccaforte R, Demers C, Baldassarre F, *et al.* Effectiveness of comprehensive disease management programmes in improving clinical outcomes in heart failure patients. A meta-analysis. *Eur J Heart Fail* 2005;**7**:1133–44

46 Rojas SV, Gagnon M-P. A systematic review of the key indicators for assessing telehomecare cost-effectiveness. *Telemed J E Health* 2008;**14**:896–904

47 Schmidt S, Schuchert A, Krieg T, *et al.* Home telemonitoring in patients with chronic heart failure: a chance to improve patient care? *Dtsch Arztebl Int* 2010;**107**:131–8

48 Tao D, Calvin KLO. Effects of self-management health information technology on glycaemic control for patients with diabet*es: a meta-analysis of randomized controlled trials. J Telemed Telecare* 2013; **19:**133-143

49 van den Berg N, Schumann M, Kraft K, *et al.* Telemedicine and telecare for older patients--a systematic review. *Maturitas* 2012;**73**:94–114

50 Whitten P, Kingsley C, Grigsby J. Results of a meta-analysis of cost-benefit research: is this a question worth asking? *J Telemed Telecare* 2000;**6 Suppl 1**:S4–6

51 Whitten PS, Mair FS, Haycox A, *et al.* Systematic review of cost effectiveness studies of telemedicine interventions. *BMJ* 2002;**324**:1434–7

52 Wootton R. Twenty years of telemedicine in chronic disease management--an evidence synthesis. *J Telemed Telecare* 2012;**18**:211–20

53 Xiang R, Li L, Liu SX. Meta-analysis and meta-regression of telehealth programmes for patients with chronic heart failure. *J Telemed Telecare* 2013;**19**:249–59

54 Whellan DJ, Hasselblad V, Peterson E, *et al.* Metaanalysis and review of heart failure disease management randomized controlled clinical trials. *Am Heart J* 2005;**149**:722–9

55 Ward AM, Takahashi O, Stevens R, *et al.* Home measurement of blood pressure and cardiovascular disease: systematic review and meta-analysis of prospective studies. *J Hypertens* 2012;**30**:449–56
