# Supplementary material for: Effects of Home Telemonitoring Interventions on Patients With Chronic Heart Failure: An Overview of Systematic Reviews
Source: J Med Internet Res. 2015 Mar 12;17(3):e63. doi: 10.2196/jmir.4174 (PMC4376138; doi:10.2196/jmir.4174)
Supplement: Supplementary file 5 [file jmir_v17i3e63_app5.pdf]

Appendix 5: Risk of Bias Assessment of the original studies included in the systematic reviews for each primary outcome of interest

All-cause mortality - Comparison 1 (Inglis et al., 2010)

Study	Random sequence generation	Allocation concealment	Outcomes assessment blinding	Incomplete outcome data (attrition bias)	Selective reporting	Study powered to detect differences	Groups comparable at baseline
Antonicelli 2008	?	?	?	+	+	+	+
Balk 2008	+	?	?	+	+	?	–
Capomolla 2004	?	?	?	?	+	?	+
Cleland 2005	+	+	?	+	+	+	?
deLusignan 2001	+	?	?	?	+	?	?
Giordano 2009	?	?	?	+	+	+	–
Goldberg 2003	?	+	+	+	?	–	+
Kielblock 2007	–	?	?	?	?	?	–
Mortara 2009	?	+	+	?	?	+	–
Soran 2008	?	?	+	?	+	+	+
Woodend 2009	?	?	?	?	–	+	?

+	**Low risk of bias**
–	**High risk of bias**
?	**Unknown risk**

All-cause mortality - Comparison 2 (Pandor et al., 2013)

Study	Random sequence generation	Allocation concealment	Outcomes assessment blinding	Incomplete outcome data (attrition bias)	Selective reporting	Study powered to detect differences	Groups comparable at baseline
Antonicelli 2008	?	?	?	+	+	+	-
Capomolla 2004	?	?	?	?	+	?	+
Cleland 2005	+	+	?	+	+	+	?
Dar 2009	+	+	?	+	+	-	+
Dendale 2011	+	?	?	+	+	+	+
Goldberg 2003	?	+	+	+	?	-	+
Kulshreshtha 2010	-	-	?	+	+	?	+
Scherr 2009	+	?	?	+	-	-	?
Woodend 2009	?	?	?	?	-	+	?

All-cause mortality - Comparison 3 (Pandor et al., 2013)

Study	Random sequence generation	Allocation concealment	Outcomes assessment blinding	Incomplete outcome data (attrition bias)	Selective reporting	Study powered to detect differences	Groups comparable at baseline
Balk 2008	+	?	?	+	+	?	–
Blum 2007	+	+	?	+	?	?	?
deLusignan 2001	+	?	?	?	+	?	?
Mortara 2009	?	+	+	?	?	+	–
Soran 2008	?	?	+	?	+	+	+
Villani 2008	+	?	?	?	?	?	+
Wade 2011	?	?	?	+	+	–	+

All-cause mortality - Comparison 4 (Pandor et al., 2013)

Study	Random sequence generation	Allocation concealment	Outcomes assessment blinding	Incomplete outcome data (attrition bias)	Selective reporting	Study powered to detect differences	Groups comparable at baseline
Giordano 2009	?	?	?	+	+	+	−
Koehler 2011	+	?	+	?	+	−	+
Zugk 2008	?	−	?	?	?	?	?

All-cause mortality - Comparison 5 (Polisena et al., 2010)

Study	Random sequence generation	Allocation concealment	Outcomes assessment blinding	Incomplete outcome data (attrition bias)	Selective reporting	Study powered to detect differences	Groups comparable at baseline
Capomolla 2004	?	?	?	?	+	?	+
Cleland 2005	+	+	?	+	+	+	?
Giordano 2009	?	?	?	+	+	+	–
Goldberg 2003	?	+	+	+	?	–	+
Schwarz 2008	+	?	?	+	+	+	?

All-cause hospitalizations - Comparison 1 (Inglis et al., 2010)

Study	Random sequence generation	Allocation concealment	Outcomes assessment blinding	Incomplete outcome data (attrition bias)	Selective reporting	Study powered to detect differences	Groups comparable at baseline
Antonicelli 2008	?	?	?	+	+	+	+
Cleland 2005	+	+	?	+	+	+	?
Giordano 2009	?	?	?	+	+	+	−
Goldberg 2003	?	+	+	+	?	−	+
Kielblock 2007	−	?	?	?	?	?	−
Mortara 2009	?	+	+	?	?	+	−
Soran 2008	?	?	+	?	+	+	+
Woodend 2009	?	?	?	?	−	+	?

All-cause hospitalizations – Comparison 2 (Pandor et al., 2013)

Study	Random sequence generation	Allocation concealment	Outcomes assessment blinding	Incomplete outcome data (attrition bias)	Selective reporting	Study powered to detect differences	Groups comparable at baseline
Antonicelli 2008	?	?	?	+	+	+	–
Cleland 2005	+	+	?	+	+	+	?
Dar 2009	+	+	?	+	+	–	+
Goldberg 2003	?	+	+	+	?	–	+
Scherr 2009	+	?	?	+	–	–	?
Woodend 2009	?	?	?	?	–	+	?

All-cause hospitalizations – Comparison 3 (Pandor et al., 2013)

Study	Random sequence generation	Allocation concealment	Outcomes assessment blinding	Incomplete outcome data (attrition bias)	Selective reporting	Study powered to detect differences	Groups comparable at baseline
Blum 2007	+	+	?	+	?	?	?
Mortara 2009	?	+	+	?	?	+	–
Soran 2008	?	?	+	?	+	+	+
Villani 2008	+	?	?	?	?	?	+
Wade 2011	?	?	?	+	+	–	+

All-cause hospitalizations – Comparison 4 (Pandor et al., 2013)

Study	Random sequence generation	Allocation concealment	Outcomes assessment blinding	Incomplete outcome data (attrition bias)	Selective reporting	Study powered to detect differences	Groups comparable at baseline
Giordano 2009	?	?	?	+	+	+	–
Koehler 2011	+	?	+	?	+	–	+
Zugk 2008	?	–	?	?	?	?	?

All-cause hospitalizations- Comparison 5 (Polisena et al., 2010)

Study	Random sequence generation	Allocation concealment	Outcomes assessment blinding	Incomplete outcome data (attrition bias)	Selective reporting	Study powered to detect differences	Groups comparable at baseline
Cleland 2005	+	+	?	+	+	+	?
Giordano 2009	?	?	?	+	+	+	–
Schwarz 2008	+	?	?	+	+	+	?

CHF-related hospitalizations - Comparison 1 (Inglis et al., 2010)

Study	Random sequence generation	Allocation concealment	Outcomes assessment blinding	Incomplete outcome data (attrition bias)	Selective reporting	Study powered to detect differences	Groups comparable at baseline
Cleland 2005	+	+	?	+	+	+	?
Giordano 2009	?	?	?	+	+	+	−
Kielblock 2007	−	?	?	?	?	?	−
Mortara 2009	?	+	+	?	?	+	−

CHF-related hospitalizations – Comparison 2 (Pandor et al., 2013)

Study	Random sequence generation	Allocation concealment	Outcomes assessment blinding	Incomplete outcome data (attrition bias)	Selective reporting	Study powered to detect differences	Groups comparable at baseline
Cleland 2005	+	+	?	+	+	+	?
Dar 2009	+	+	?	+	+	–	+
Goldberg 2003	?	+	+	+	?	–	+

CHF-related hospitalizations – Comparison 3 (Pandor et al., 2013)

Study	Random sequence generation	Allocation concealment	Outcomes assessment blinding	Incomplete outcome data (attrition bias)	Selective reporting	Study powered to detect differences	Groups comparable at baseline
Mortara 2009	?	+	+	?	?	+	–
Villani 2008	+	?	?	?	?	?	+

CHF-related hospitalizations – Comparison 3 (Pandor et al., 2013)

Study	Random sequence generation	Allocation concealment	Outcomes assessment blinding	Incomplete outcome data (attrition bias)	Selective reporting	Study powered to detect differences	Groups comparable at baseline
Giordano 2009	?	?	?	+	+	+	–
Koehler 2011	+	?	+	?	+	–	+
Zugk 2008	?	–	?	?	?	?	?
