# Supplementary material for: Effects of Home Telemonitoring Interventions on Patients With Chronic Heart Failure: An Overview of Systematic Reviews
Source: J Med Internet Res. 2015 Mar 12;17(3):e63. doi: 10.2196/jmir.4174 (PMC4376138; doi:10.2196/jmir.4174)

Analysis 1.1.: Comparison 1 Automated device-based telemonitoring (TM and TM+) vs. Usual Care; Outcome 1: All-cause mortality

Study or Subgroup	Experimental Events	Experimental Total	Control Events	Control Total	Weight	Risk Ratio M-H, Random, 95% CI	Year
Goldberg 2003	11	138	26	142	8.5%	0.44 [0.22, 0.85]	2003
Cleland 2005	28	168	20	85	14.4%	0.71 [0.42, 1.18]	2005
Blum 2007	17	102	21	102	11.3%	0.81 [0.45, 1.44]	2007
Kielblock 2007	37	251	69	251	29.2%	0.54 [0.37, 0.77]	2007
Balk 2008	9	101	8	113	4.5%	1.26 [0.50, 3.14]	2008
Schwarz 2008	4	44	7	40	2.8%	0.52 [0.16, 1.64]	2008
Soran 2008	11	160	17	155	7.2%	0.63 [0.30, 1.29]	2008
Zugk 2008	3	58	1	30	0.8%	1.55 [0.17, 14.28]	2008
Antonicelli 2008	3	28	5	29	2.1%	0.62 [0.16, 2.36]	2008
Giordano 2009	21	230	32	230	14.0%	0.66 [0.39, 1.10]	2009
Kulshreshtha 2010	4	42	4	68	2.1%	1.62 [0.43, 6.13]	2010
Wade 2011	6	164	6	152	3.1%	0.93 [0.31, 2.81]	2011
**Total (95% CI)**		**1486**		**1397**	**100.0%**	**0.65 [0.54, 0.79]**	
Total events	154		216				

Heterogeneity: Tau² = 0.00; Chi² = 8.12, df = 11 (P = 0.70); I² = 0%
Test for overall effect: Z = 4.30 (P < 0.0001)

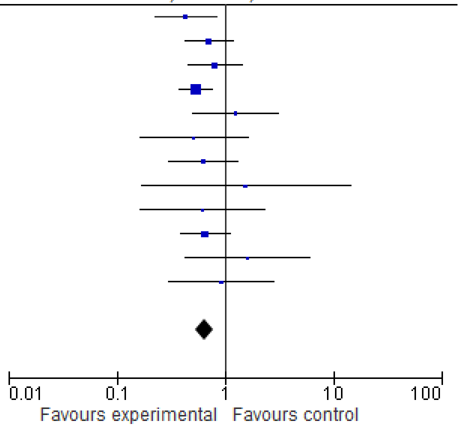

Analysis 1.2.: Comparison 1 Automated device-based telemonitoring (TM and TM+) vs. Usual Care; Outcome 2: All-cause hospitalizations

Study or Subgroup	Experimental Events	Experimental Total	Control Events	Control Total	Weight	Risk Ratio M-H, Random, 95% CI	Year
Goldberg 2003	65	138	67	142	12.1%	1.00 [0.78, 1.28]	2003
Cleland 2005	80	168	46	85	12.0%	0.88 [0.68, 1.13]	2005
Blum 2007	42	102	28	102	8.4%	1.50 [1.01, 2.22]	2007
Kielblock 2007	157	251	176	251	15.6%	0.89 [0.79, 1.01]	2007
Antonicelli 2008	9	28	26	29	5.6%	0.36 [0.21, 0.62]	2008
Schwarz 2008	12	44	13	40	4.3%	0.84 [0.43, 1.62]	2008
Soran 2008	75	160	66	155	12.2%	1.10 [0.86, 1.41]	2008
Zugk 2008	24	58	17	30	7.4%	0.73 [0.47, 1.13]	2008
Giordano 2009	67	230	96	230	12.0%	0.70 [0.54, 0.90]	2009
Wade 2011	57	164	49	152	10.3%	1.08 [0.79, 1.47]	2011
**Total (95% CI)**		**1343**		**1216**	**100.0%**	**0.89 [0.76, 1.05]**	
Total events	588		584				

Heterogeneity: Tau² = 0.04; Chi² = 26.58, df = 9 (P = 0.002); I² = 66%
Test for overall effect: Z = 1.38 (P = 0.17)

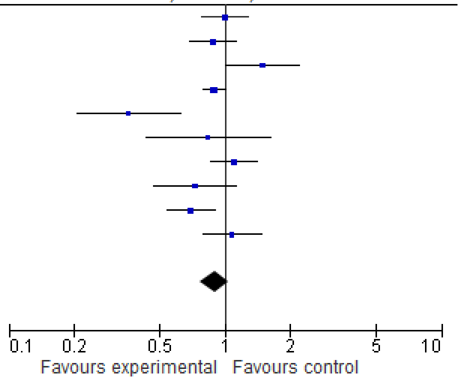

Analysis 1.3.: Comparison 1 Automated device-based telemonitoring (TM and TM+) vs. Usual Care; Outcome 3: HF hospitalizations

Study or Subgroup	Experimental Events	Experimental Total	Control Events	Control Total	Weight	Risk Ratio M-H, Random, 95% CI	Year
Goldberg 2003	71	251	82	251	27.9%	0.87 [0.66, 1.13]	2003
Cleland 2005	40	168	24	85	13.4%	0.84 [0.55, 1.30]	2005
Kielblock 2007	71	251	82	251	27.9%	0.87 [0.66, 1.13]	2007
Zugk 2008	18	58	16	30	10.2%	0.58 [0.35, 0.97]	2008
Giordano 2009	43	230	73	230	20.6%	0.59 [0.42, 0.82]	2009
**Total (95% CI)**		**958**		**847**	**100.0%**	**0.77 [0.64, 0.91]**	
Total events	243		277				

Heterogeneity: Tau² = 0.01; Chi² = 5.37, df = 4 (P = 0.25); I² = 25%
Test for overall effect: Z = 3.01 (P = 0.003)

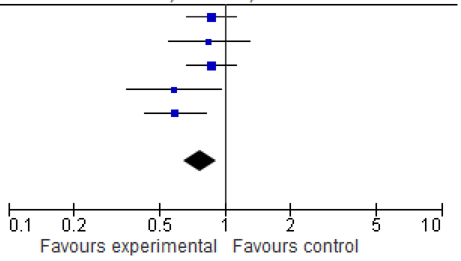

Analysis 2.1.: Comparison 2 Telemonitoring of vital signs (TM) vs. Usual Care; Outcome 1: All-cause mortality

Study or Subgroup	Experimental Events	Experimental Total	Control Events	Control Total	Weight	Risk Ratio M-H, Random, 95% CI	Year
Cleland 2005	28	168	20	85	20.1%	0.71 [0.42, 1.18]	2005
Kielblock 2007	37	251	69	251	40.7%	0.54 [0.37, 0.77]	2007
Blum 2007	17	102	21	102	15.7%	0.81 [0.45, 1.44]	2007
Zugk 2008	3	58	1	30	1.1%	1.55 [0.17, 14.28]	2008
Antonicelli 2008	3	28	5	29	2.9%	0.62 [0.16, 2.36]	2008
Giordano 2009	21	230	32	230	19.5%	0.66 [0.39, 1.10]	2009
**Total (95% CI)**		**837**		**727**	**100.0%**	**0.64 [0.51, 0.80]**	
Total events	109		148				

Heterogeneity: Tau² = 0.00; Chi² = 2.34, df = 5 (P = 0.80); I² = 0%
Test for overall effect: Z = 3.83 (P = 0.0001)

Risk Ratio M-H, Random, 95% CI

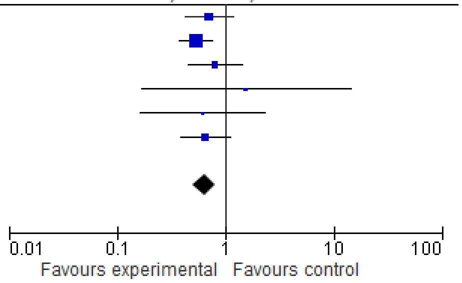

Analysis 2.2.: Comparison 2 Telemonitoring of vital signs (TM) vs. Usual Care; Outcome 2: All-cause hospitalizations

Study or Subgroup	Experimental Events	Experimental Total	Control Events	Control Total	Weight	Risk Ratio M-H, Random, 95% CI	Year
Cleland 2005	80	168	46	85	19.2%	0.88 [0.68, 1.13]	2005
Kielblock 2007	157	251	176	251	22.9%	0.89 [0.79, 1.01]	2007
Blum 2007	42	102	28	102	14.7%	1.50 [1.01, 2.22]	2007
Zugk 2008	24	58	17	30	13.4%	0.73 [0.47, 1.13]	2008
Antonicelli 2008	9	28	26	29	10.6%	0.36 [0.21, 0.62]	2008
Giordano 2009	67	230	96	230	19.2%	0.70 [0.54, 0.90]	2009
**Total (95% CI)**		**837**		**727**	**100.0%**	**0.81 [0.64, 1.03]**	
Total events	379		389				

Heterogeneity: Tau² = 0.06; Chi² = 20.96, df = 5 (P = 0.0008); I² = 76%
Test for overall effect: Z = 1.73 (P = 0.08)

Risk Ratio M-H, Random, 95% CI

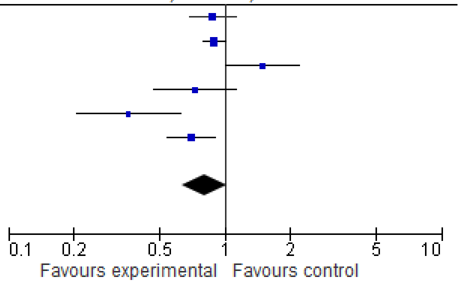

Analysis 2.3.: Comparison 2 Telemonitoring of vital signs (TM) vs. Usual Care; Outcome 3: HF hospitalizations

Study or Subgroup	Experimental Events	Experimental Total	Control Events	Control Total	Weight	Risk Ratio M-H, Random, 95% CI	Year
Cleland 2005	40	168	24	85	19.5%	0.84 [0.55, 1.30]	2005
Kielblock 2007	71	251	82	251	36.8%	0.87 [0.66, 1.13]	2007
Zugk 2008	18	58	16	30	15.2%	0.58 [0.35, 0.97]	2008
Giordano 2009	43	230	73	230	28.6%	0.59 [0.42, 0.82]	2009
**Total (95% CI)**		**707**		**596**	**100.0%**	**0.73 [0.58, 0.91]**	
Total events	172		195				

Heterogeneity: Tau² = 0.02; Chi² = 4.39, df = 3 (P = 0.22); I² = 32%
Test for overall effect: Z = 2.84 (P = 0.005)

Risk Ratio M-H, Random, 95% CI

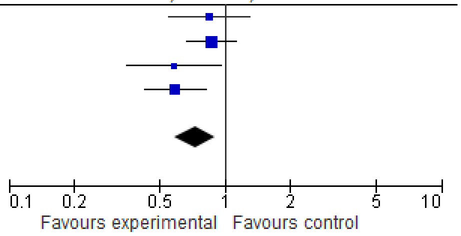

Analysis 3.1.: Comparison 3 Telemonitoring of vital signs and symptoms (TM+) vs. Usual Care; Outcome 1: All-cause mortality

	Experimental		Control			Risk Ratio		Risk Ratio
Study or Subgroup	Events	Total	Events	Total	Weight	M-H, Random, 95% CI	Year	M-H, Random, 95% CI
Goldberg 2003	11	138	26	142	28.2%	0.44 [0.22, 0.85]	2003	
Balk 2008	9	101	8	113	16.5%	1.26 [0.50, 3.14]	2008	
Schwarz 2008	4	44	7	40	10.9%	0.52 [0.16, 1.64]	2008	
Soran 2008	11	160	17	155	24.5%	0.63 [0.30, 1.29]	2008	
Kulshreshtha 2010	4	42	4	68	8.3%	1.62 [0.43, 6.13]	2010	
Wade 2011	6	164	6	152	11.6%	0.93 [0.31, 2.81]	2011	
**Total (95% CI)**		**649**		**670**	**100.0%**	**0.70 [0.47, 1.04]**		
Total events	45		68					

Heterogeneity: Tau² = 0.03; Chi² = 5.66, df = 5 (P = 0.34); I² = 12%
Test for overall effect: Z = 1.74 (P = 0.08)

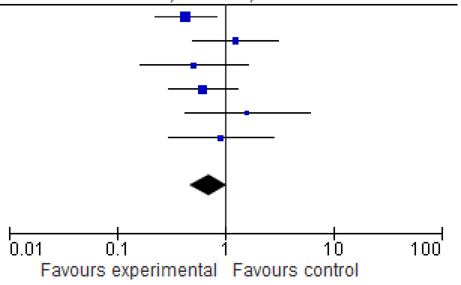

Analysis 3.2.: Comparison 3 Telemonitoring of vital signs and symptoms (TM+) vs. Usual Care; Outcome 2: All-cause hospitalizations

	Experimental		Control			Risk Ratio	Risk Ratio
Study or Subgroup	Events	Total	Events	Total	Weight	M-H, Random, 95% CI	M-H, Random, 95% CI
Goldberg 2003	65	138	67	142	35.8%	1.00 [0.78, 1.28]	
Schwarz 2008	12	44	13	40	5.1%	0.84 [0.43, 1.62]	
Soran 2008	75	160	66	155	36.4%	1.10 [0.86, 1.41]	
Wade 2011	57	164	49	152	22.7%	1.08 [0.79, 1.47]	
**Total (95% CI)**		**506**		**489**	**100.0%**	**1.04 [0.90, 1.21]**	
Total events	209		195				

Heterogeneity: Tau² = 0.00; Chi² = 0.77, df = 3 (P = 0.86); I² = 0%
Test for overall effect: Z = 0.56 (P = 0.58)

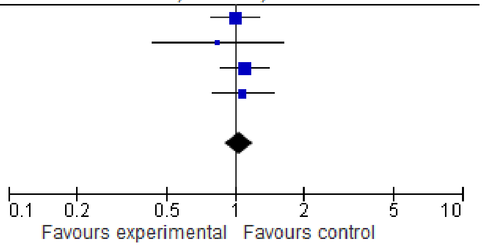

Analysis 3.3.: Comparison 3 Telemonitoring of vital signs and symptoms (TM+) vs. Usual Care; Outcome 3: HF hospitalizations

	Experimental		Control			Risk Ratio		Risk Ratio
Study or Subgroup	Events	Total	Events	Total	Weight	M-H, Random, 95% CI	Year	M-H, Random, 95% CI
Goldberg 2003	71	251	82	251	100.0%	0.87 [0.66, 1.13]	2003	
**Total (95% CI)**		**251**		**251**	**100.0%**	**0.87 [0.66, 1.13]**		
Total events	71		82					

Heterogeneity: Not applicable
Test for overall effect: Z = 1.06 (P = 0.29)

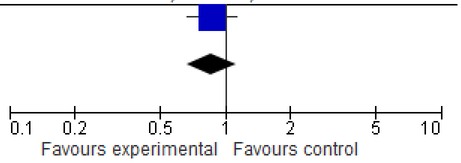

Analysis 4.1.: Comparison 4 Mobile Telemonitoring MT" vs. Usual Care; Outcome 1: All-cause mortality

	Experimental		Control			Risk Ratio		Risk Ratio
Study or Subgroup	Events	Total	Events	Total	Weight	M-H, Random, 95% CI	Year	M-H, Random, 95% CI
Villani 2008	5	33	9	44	24.1%	0.74 [0.27, 2.00]	2008	
Scherr 2009	0	66	1	54	3.7%	0.27 [0.01, 6.58]	2009	
Koehler 2011	54	354	55	356	50.1%	0.99 [0.70, 1.39]	2011	
Dendale 2011	4	80	14	80	22.1%	0.29 [0.10, 0.83]	2011	
**Total (95% CI)**		**533**		**534**	**100.0%**	**0.67 [0.35, 1.26]**		
Total events	63		79					

Heterogeneity: Tau² = 0.18; Chi² = 5.35, df = 3 (P = 0.15); I² = 44%
Test for overall effect: Z = 1.25 (P = 0.21)

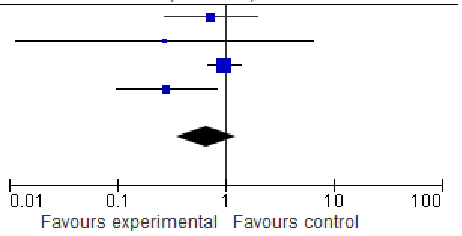

Analysis 4.2.: Comparison 4 "MT" vs. Usual Care; Outcome 2: All-cause hospitalizations

	Experimental		Control			Risk Ratio		Risk Ratio
Study or Subgroup	Events	Total	Events	Total	Weight	M-H, Random, 95% CI	Year	M-H, Random, 95% CI
Villani 2008	23	33	28	44	33.5%	1.10 [0.80, 1.50]	2008	
Scherr 2009	11	66	17	54	12.9%	0.53 [0.27, 1.03]	2009	
Koehler 2011	192	354	179	356	53.6%	1.08 [0.94, 1.24]	2011	
**Total (95% CI)**		**453**		**454**	**100.0%**	**0.99 [0.76, 1.29]**		
Total events	226		224					

Heterogeneity: Tau² = 0.03; Chi² = 4.33, df = 2 (P = 0.11); I² = 54%
Test for overall effect: Z = 0.08 (P = 0.94)

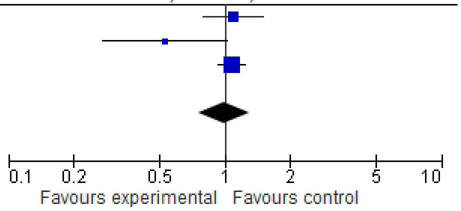

Analysis 4.3.: Comparison 4 "MT" vs. Usual Care; Outcome 3: HF hospitalizations

	Experimental		Control			Risk Ratio		Risk Ratio
Study or Subgroup	Events	Total	Events	Total	Weight	M-H, Random, 95% CI	Year	M-H, Random, 95% CI
Villani 2008	6	33	17	44	30.3%	0.47 [0.21, 1.06]	2008	
Koehler 2011	64	354	74	356	69.7%	0.87 [0.64, 1.17]	2011	
**Total (95% CI)**		**387**		**400**	**100.0%**	**0.72 [0.42, 1.26]**		
Total events	70		91					

Heterogeneity: Tau² = 0.09; Chi² = 1.93, df = 1 (P = 0.17); I² = 48%
Test for overall effect: Z = 1.15 (P = 0.25)

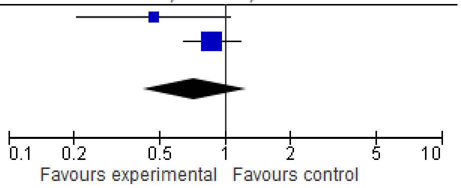

Analysis 5.1.: Comparison 4 Interactive Voice Response (IVR) vs. Usual Care; Outcome 1: All-cause mortality

Study or Subgroup	Experimental Events	Experimental Total	Control Events	Control Total	Weight	Risk Ratio M-H, Random, 95% CI
Capomolla 2004	5	67	7	66	34.7%	0.70 [0.24, 2.11]
Mortara 2009	15	195	9	160	65.3%	1.37 [0.61, 3.04]
**Total (95% CI)**		**262**		**226**	**100.0%**	**1.09 [0.57, 2.07]**
Total events	20		16			

Heterogeneity: Tau² = 0.00; Chi² = 0.92, df = 1 (P = 0.34); I² = 0%
Test for overall effect: Z = 0.25 (P = 0.80)

Risk Ratio M-H, Random, 95% CI

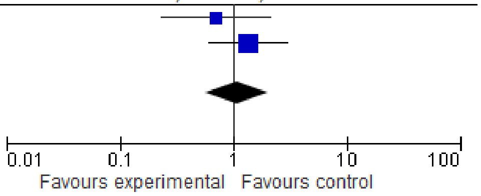

Analysis 5.2.: Comparison 4 Interactive Voice Response (IVR) vs. Usual Care; Outcome 2: All-cause hospitalizations

Study or Subgroup	Experimental Events	Experimental Total	Control Events	Control Total	Weight	Risk Ratio M-H, Random, 95% CI
Mortara 2009	69	195	48	160	100.0%	1.18 [0.87, 1.60]
**Total (95% CI)**		**195**		**160**	**100.0%**	**1.18 [0.87, 1.60]**
Total events	69		48			

Heterogeneity: Not applicable
Test for overall effect: Z = 1.07 (P = 0.29)

Risk Ratio M-H, Random, 95% CI

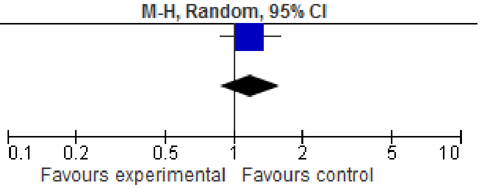

Analysis 5.3.: Comparison 4 Interactive Voice Response (IVR) vs. Usual Care; Outcome 3: HF hospitalizations

Study or Subgroup	Experimental Events	Experimental Total	Control Events	Control Total	Weight	Risk Ratio M-H, Random, 95% CI	Year
Mortara 2009	35	195	28	160	100.0%	1.03 [0.65, 1.61]	2009
**Total (95% CI)**		**195**		**160**	**100.0%**	**1.03 [0.65, 1.61]**	
Total events	35		28				

Heterogeneity: Not applicable
Test for overall effect: Z = 0.11 (P = 0.91)

Risk Ratio M-H, Random, 95% CI

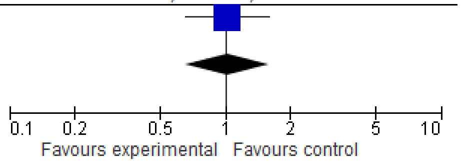

Analysis 6.1.: Comparison 5 Video-consultation with vital signs monitoring (VC+) vs. Usual Care; Outcome 1: All-cause mortality

Study or Subgroup	Experimental Events	Experimental Total	Control Events	Control Total	Weight	Risk Ratio M-H, Random, 95% CI
de Lusignan 2001	2	10	3	10	39.7%	0.67 [0.14, 3.17]
Woodend 2008	5	62	4	59	60.3%	1.19 [0.34, 4.22]
**Total (95% CI)**		**72**		**69**	**100.0%**	**0.95 [0.35, 2.53]**
Total events	7		7			

Heterogeneity: Tau² = 0.00; Chi² = 0.32, df = 1 (P = 0.57); I² = 0%
Test for overall effect: Z = 0.11 (P = 0.91)

Risk Ratio
M-H, Random, 95% CI

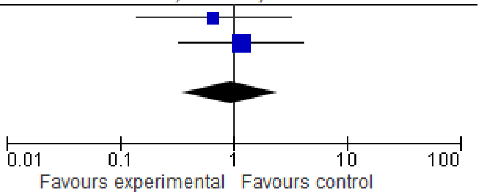

Analysis 6.2.: Comparison 5 Video-consultation with vital signs monitoring (VC+) vs. Usual Care; Outcome 2: All-cause hospitalizations

Study or Subgroup	Experimental Events	Experimental Total	Control Events	Control Total	Weight	Risk Ratio M-H, Random, 95% CI
Woodend 2008	60	62	54	59	100.0%	1.06 [0.97, 1.16]
**Total (95% CI)**		**62**		**59**	**100.0%**	**1.06 [0.97, 1.16]**
Total events	60		54			

Heterogeneity: Not applicable
Test for overall effect: Z = 1.21 (P = 0.22)

Risk Ratio
M-H, Random, 95% CI

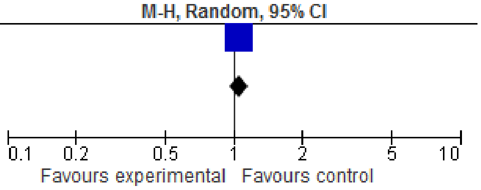

Analysis 6.3.: Comparison 5 Video-consultation with vital signs monitoring (VC+) vs. Usual Care; Outcome 3: HF hospitalizations

No results reported for this outcome

Analysis 7.1.: Comparison 7 All types of HT interventions vs. Usual Care; Outcome 1: All-cause mortality

Study or Subgroup	Experimental Events	Experimental Total	Control Events	Control Total	Weight	Risk Ratio M-H, Random, 95% CI	Year
de Lusignan 2001	2	10	3	10	1.0%	0.67 [0.14, 3.17]	2001
Goldberg 2003	11	138	26	142	5.6%	0.44 [0.22, 0.85]	2003
Capomolla 2004	5	67	7	66	2.1%	0.70 [0.24, 2.11]	2004
Cleland 2005	28	168	20	85	9.5%	0.71 [0.42, 1.18]	2005
Blum 2007	17	102	21	102	7.4%	0.81 [0.45, 1.44]	2007
Kielblock 2007	37	251	69	251	19.2%	0.54 [0.37, 0.77]	2007
Balk 2008	9	101	8	113	3.0%	1.26 [0.50, 3.14]	2008
Antonicelli 2008	3	28	5	29	1.4%	0.62 [0.16, 2.36]	2008
Villani 2008	5	33	9	44	2.5%	0.74 [0.27, 2.00]	2008
Woodend 2008	5	62	4	59	1.5%	1.19 [0.34, 4.22]	2008
Zugk 2008	3	58	1	30	0.5%	1.55 [0.17, 14.28]	2008
Schwarz 2008	4	44	7	40	1.9%	0.52 [0.16, 1.64]	2008
Soran 2008	11	160	17	155	4.7%	0.63 [0.30, 1.29]	2008
Giordano 2009	21	230	32	230	9.2%	0.66 [0.39, 1.10]	2009
Mortara 2009	15	195	9	160	3.9%	1.37 [0.61, 3.04]	2009
Scherr 2009	0	66	1	54	0.2%	0.27 [0.01, 6.58]	2009
Kulshreshtha 2010	4	42	4	68	1.4%	1.62 [0.43, 6.13]	2010
Dendale 2011	4	80	14	80	2.2%	0.29 [0.10, 0.83]	2011
Wade 2011	6	164	6	152	2.0%	0.93 [0.31, 2.81]	2011
Koehler 2011	54	354	55	356	20.8%	0.99 [0.70, 1.39]	2011
**Total (95% CI)**		**2353**		**2226**	**100.0%**	**0.73 [0.62, 0.85]**	
Total events	244		318				

Heterogeneity: Tau² = 0.00; Chi² = 18.61, df = 19 (P = 0.48); I² = 0%

Test for overall effect: Z = 3.95 (P < 0.0001)

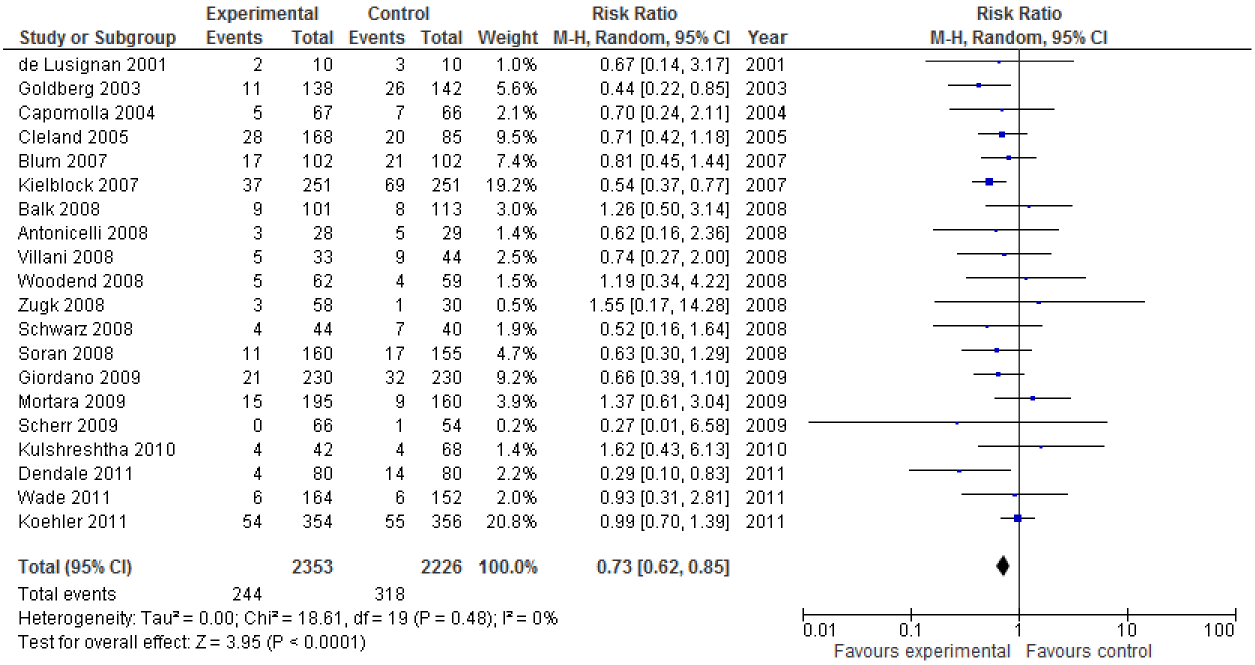

Analysis 7.2.: Comparison 7 All types of HT interventions vs. Usual Care; Outcome 2: All-cause hospitalizations

Study or Subgroup	Experimental Events	Experimental Total	Control Events	Control Total	Weight	Risk Ratio M-H, Random, 95% CI	Year
Goldberg 2003	65	138	67	142	7.8%	1.00 [0.78, 1.28]	2003
Cleland 2005	80	168	46	85	7.7%	0.88 [0.68, 1.13]	2005
Kielblock 2007	157	251	176	251	11.0%	0.89 [0.79, 1.01]	2007
Blum 2007	42	102	28	102	4.9%	1.50 [1.01, 2.22]	2007
Zugk 2008	24	58	17	30	4.2%	0.73 [0.47, 1.13]	2008
Antonicelli 2008	9	28	26	29	3.0%	0.36 [0.21, 0.62]	2008
Schwarz 2008	12	44	13	40	2.3%	0.84 [0.43, 1.62]	2008
Villani 2008	23	33	28	44	6.2%	1.10 [0.80, 1.50]	2008
Soran 2008	75	160	66	155	7.8%	1.10 [0.86, 1.41]	2008
Woodend 2008	60	62	54	59	11.8%	1.06 [0.97, 1.16]	2008
Giordano 2009	67	230	96	230	7.6%	0.70 [0.54, 0.90]	2009
Scherr 2009	11	66	17	54	2.2%	0.53 [0.27, 1.03]	2009
Mortara 2009	69	195	48	160	6.5%	1.18 [0.87, 1.60]	2009
Wade 2011	57	164	49	152	6.3%	1.08 [0.79, 1.47]	2011
Koehler 2011	192	354	179	356	10.6%	1.08 [0.94, 1.24]	2011
**Total (95% CI)**		**2053**		**1889**	**100.0%**	**0.95 [0.85, 1.06]**	
Total events	943		910				

Heterogeneity: Tau² = 0.02; Chi² = 40.92, df = 14 (P = 0.0002); I² = 66%

Test for overall effect: Z = 0.88 (P = 0.38)

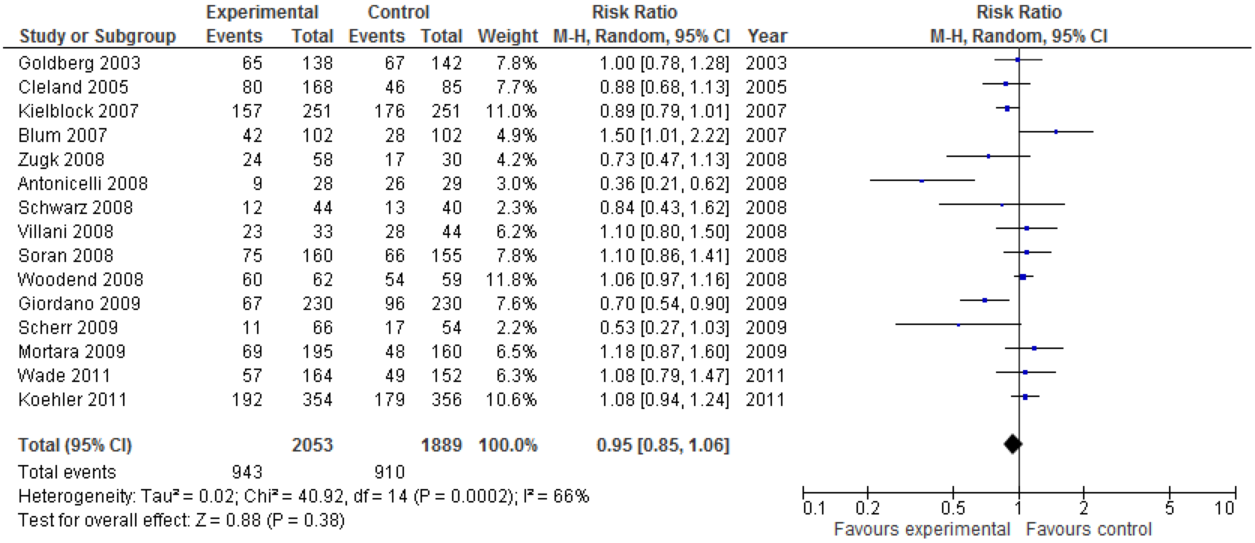

Analysis 7.3.: Comparison 7 All types of HT interventions vs. Usual Care; Outcome 3: HF hospitalizations

	Experimental		Control			Risk Ratio	
Study or Subgroup	Events	Total	Events	Total	Weight	M-H, Random, 95% CI	Year
Goldberg 2003	71	251	82	251	20.2%	0.87 [0.66, 1.13]	2003
Cleland 2005	40	168	24	85	9.3%	0.84 [0.55, 1.30]	2005
Kielblock 2007	71	251	82	251	20.2%	0.87 [0.66, 1.13]	2007
Zugk 2008	18	58	16	30	7.0%	0.58 [0.35, 0.97]	2008
Villani 2008	6	33	17	44	2.9%	0.47 [0.21, 1.06]	2008
Mortara 2009	35	195	28	160	8.7%	1.03 [0.65, 1.61]	2009
Giordano 2009	43	230	73	230	14.7%	0.59 [0.42, 0.82]	2009
Koehler 2011	64	354	74	356	16.9%	0.87 [0.64, 1.17]	2011
**Total (95% CI)**		**1540**		**1407**	**100.0%**	**0.79 [0.69, 0.91]**	
Total events	348		396				

Heterogeneity: Tau² = 0.01; Chi² = 8.65, df = 7 (P = 0.28); I² = 19%

Test for overall effect: Z = 3.20 (P = 0.001)

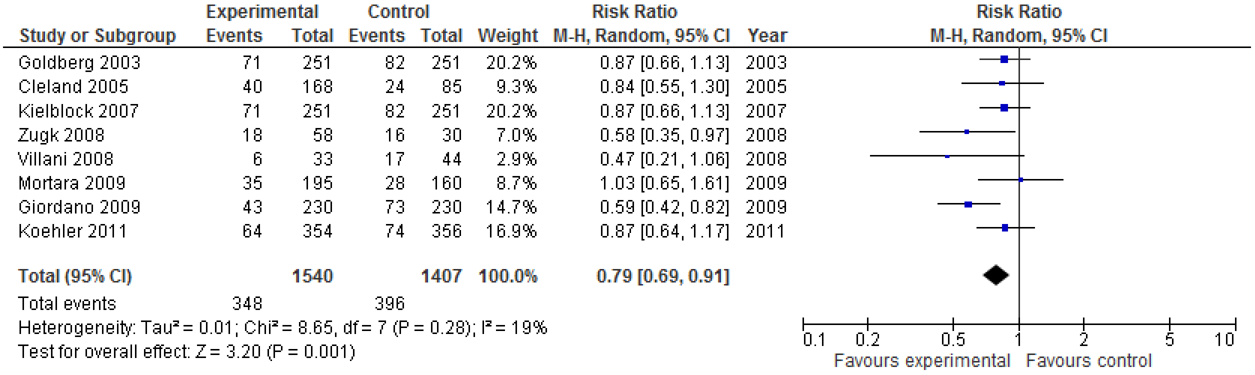

Supplement: Supplementary file 6 [file jmir_v17i3e63_app6.pdf]
